# A Synthesis of Tagging Studies Examining the Behaviour and Survival of Anadromous Salmonids in Marine Environments

**DOI:** 10.1371/journal.pone.0031311

**Published:** 2012-03-14

**Authors:** S. Matthew Drenner, Timothy D. Clark, Charlotte K. Whitney, Eduardo G. Martins, Steven J. Cooke, Scott G. Hinch

**Affiliations:** 1 Pacific Salmon Ecology and Conservation Laboratory, Department of Forest Sciences, University of British Columbia, Vancouver, British Columbia, Canada; 2 Australian Institute of Marine Science, Townsville MC, Townsville, Queensland, Australia; 3 Fish Ecology and Conservation Physiology Laboratory, Department of Biology and Institute of Environmental Science, Carleton University, Ottawa, Ontario, Canada; University of California Davis, United States of America

## Abstract

This paper synthesizes tagging studies to highlight the current state of knowledge concerning the behaviour and survival of anadromous salmonids in the marine environment. Scientific literature was reviewed to quantify the number and type of studies that have investigated behaviour and survival of anadromous forms of Pacific salmon (*Oncorhynchus* spp.), Atlantic salmon (*Salmo salar*), brown trout (*Salmo trutta*), steelhead (*Oncorhynchus mykiss*), and cutthroat trout (*Oncorhynchus clarkii*). We examined three categories of tags including electronic (e.g. acoustic, radio, archival), passive (e.g. external marks, Carlin, coded wire, passive integrated transponder [PIT]), and biological (e.g. otolith, genetic, scale, parasites). Based on 207 papers, survival rates and behaviour in marine environments were found to be extremely variable spatially and temporally, with some of the most influential factors being temperature, population, physiological state, and fish size. Salmonids at all life stages were consistently found to swim at an average speed of approximately one body length per second, which likely corresponds with the speed at which transport costs are minimal. We found that there is relatively little research conducted on open-ocean migrating salmonids, and some species (e.g. masu [*O. masou*] and amago [*O. rhodurus*]) are underrepresented in the literature. The most common forms of tagging used across life stages were various forms of external tags, coded wire tags, and acoustic tags, however, the majority of studies did not measure tagging/handling effects on the fish, tag loss/failure, or tag detection probabilities when estimating survival. Through the interdisciplinary application of existing and novel technologies, future research examining the behaviour and survival of anadromous salmonids could incorporate important drivers such as oceanography, tagging/handling effects, predation, and physiology.

## Introduction

### Importance of salmonids, and recent population trends

Anadromous salmonids are important ecologically, culturally, and economically across the globe, as a critical aspect of their ecological systems, as a significant commercial and artisanal fishery, and as a sensitive environmental indicator. They provide cultural and social value to local and native peoples [1], and form a multi-million dollar global fishery. While at sea and in freshwater, salmonids are important prey items and nutrient sources, and they continue to provide such benefits after death by supplying enrichment to terrestrial systems as they decay on the riverbed [2], [3]. An anadromous life history means that salmonids can be affected by changes in both freshwater and marine ecosystems, including widespread habitat degradation, altered ecosystem productivity, overharvest, and climate change [4]–[10].

Over the last century, many populations of wild salmonids have declined in abundance [7], [11]–[15]. In recent years, some populations have been threatened with extinction and extirpation [16], [17] resulting in many areas that are either devoid of salmon [8], [18] or are reliant on hatchery-raised salmon populations [15]. In many regions, enhancement programs such as hatcheries and fish farms (aquaculture) have been introduced in an attempt to supplement wild populations and to meet the global demands for human consumption of salmon. However, hatchery and aquaculture enhancement may have inadvertently introduced a new suite of concerns for wild populations, such as interbreeding risk resulting in a loss of genetic variation, increased competition for scarce resources and habitat, and an increase in disease prevalence and dispersal [19]–[22]. The ‘crisis’ of declining salmon populations is currently considered one of the major issues in fisheries biology [8], and extensive management efforts are being applied in an attempt to conserve at-risk populations. In general, population declines seem to be more drastic in southern latitudes, and are less apparent at higher latitudes [23]–[26]. Perhaps the most alarming aspect is that the causes for the declines remain largely unknown.

The majority of research and management efforts on anadromous salmonids have historically focused on the freshwater phase of the lifecycle (including outmigrating juveniles, and upriver migrating adults) [3], [5]. The reason for this is largely technical, as there are inherent difficulties with studying salmonids in the marine environment. Consequently, current knowledge of the marine phase of the lifecycle (i.e. both juveniles and adults in saline environments including estuaries, coastal waters and open-ocean) is still quite limited, despite it being generally acknowledged as a critical stage related to low survival caused by both abiotic and biotic factors [7], [23], [27]–[30].

### Objectives of this review

In light of the declining abundance of many wild salmon populations, and the knowledge gap relating to the behaviour and survival of salmonids in the marine environment, this paper has three main objectives. First, we reviewed the scientific literature to quantify the number and range of studies that have investigated aspects of salmon behaviour and survival in the marine environment. Owing to the impressive, informative and long-term data sets that have been generated through tagging studies, we focused our literature review on studies that utilized some form of tagging to investigate aspects of salmonid biology in marine waters. Second, we sought to synthesize the current state of knowledge concerning salmonid behaviour and survival in the marine environment. Third, we highlight particular knowledge gaps that require further attention and suggest some approaches, both technological and methodological, from which future studies could benefit in order to improve our understanding of salmonid biology. The review is broken into various life history phases that occur within the marine environment, namely the out-migration of juveniles (plus Atlantic salmon kelts [*Salmo salar*] and adult steelhead [*Oncorhynchus mykiss*]), sub-adults and adults in the open-ocean, and mature adults on their return spawning migration towards freshwater. To fully appreciate the complexity of the ‘salmon crisis’, with an aim to target key factors that may be responsible for the global decline in abundance of wild salmon, we first examine the complex life histories of the salmonids, followed by a brief review of tag types that are commonly applied to salmonids.

### Life histories of salmonids

There is only one species of Atlantic salmon (family Salmonidae; species *Salmo salar*), while the Pacific salmonids (family Salmonidae; genus *Oncorhynchus*) comprise eight species, including Chinook (*O. tshawytscha*), chum (*O. keta*), coho (*O. kisutch*), pink (*O. gorbuscha*), sockeye (*O. nerka*), masu (*O. masou*), amago (*O. rhodurus*), and steelhead (*O. mykiss*). In addition, there are anadromous forms of brown trout and sea trout (*S. trutta*), and cutthroat trout (*O. clarkii*). Various species of Pacific salmon are found on both sides of the northern Pacific Ocean (Western Canada and the U.S. from California to Alaska, Japan, Russia, and Korea), whereas Atlantic salmon are found in the north-western (Spain north to the British Isles, Greenland, Norway and Finland) and the north-eastern (eastern Canada and the U.S.) Atlantic Ocean. Both Pacific and Atlantic salmon are considered anadromous, but in many of the species there are minorities of non-anadromous forms that remain in freshwater for the duration of their lives, however the latter are not included in this review.

There is a tremendous amount of variation in the timing of different life stages between and within anadromous salmon species (see for Pacific salmon and trout: [3], [31]; for Atlantic salmon: [32], [33]). However, most anadromous salmonids can be characterized by a generalized life cycle. Adults of both Atlantic and Pacific salmon spawn in freshwater streams or lakes (and some in intertidal areas; pink and chum salmon) and either die soon after (semelparous species of Pacific salmon), or have the ability to survive the spawning period (i.e. iteroparous species). Eggs deposited in substrate hatch to produce alevins, which remain under gravel and use a yolk sac for nutrition until they emerge as fry four to six weeks later. At this point, some species migrate directly to the ocean, while others remain in freshwater as parr and feed on small aquatic organisms typically for one to two years before migrating to the ocean. In the spring of a subsequent year, fish still in freshwater become smolts and migrate to the sea to forage and mature for a number of years before returning to natal spawning grounds to reproduce. The return spawning migration is among the most spectacular in the animal kingdom, with some species traversing entire oceans before entering freshwater and migrating up to 1,500 km upriver to spawn [5], [31].

### Overview of tagging technologies and techniques

Various types of tags have historically, and are currently used for research on salmonids. Tags can be grouped into three main categories: passive, electronic, and biological. Passive tags are those which do not have an inbuilt battery, they often involve a visual marking of the fish, and they are primarily used for identification of individuals or groups once they are recaptured or within sight. Passive tags include external marks (e.g. adipose fin clips), external visual tags ( = t-bar anchor tags [e.g. Carlin, Floy, Peterson Disk, cinch tags]) and internally injected tags such as coded wire tags (CWT) and passive integrated transponder (PIT) tags. While PIT tags are characterised as passive, they use radio frequency energy from an antenna or a closely held scanner to power the tag circuits and allow a unique identifying signal to be transmitted.

Electronic tags (reviewed in [34]) were characterised as those which possess an inbuilt battery and may either store acquired data to an onboard memory chip [e.g. archival tags ( = data loggers)] or transmit the data, typically via acoustic or radio transmission, to a nearby receiver (e.g. standard acoustic and radio tags). There exist combined technology tags, such as pop-off satellite tags (PSATs) and smart position or temperature transmitting tags (SPOTs), which first archive and then transmit data to a satellite. Electronic tags have been used to measure a great range of environmental, behavioural and physiological information from fish, including temperature, depth, light, global or local position, acceleration, swimming muscle contractions, and heart rate [35]–[41]. Radio signals attenuate rapidly in saltwater, so radio tags are typically restricted to freshwater environments or when the radio signal can transmit through air such as with PSATs or SPOT tags. Acoustic tags, whether manually tracked by boat or automatically by an array of installed receivers, have proven useful in both marine and freshwater environments, although signal transmission can be affected by water depth and extraneous acoustic noise. Electronic tags are typically several orders of magnitude larger and more expensive than passive tags, which can both lower sample sizes within a study and restrict tagging to large individuals. Electronic tags that transmit allow for tracking along a migration route, meaning that tag recovery is not necessary to obtain data. Archival tags can acquire data even when fish are not within range of a receiver, but they must be recovered to download stored data.

Biological tags, or ‘natural tags’, include natural distinguishable markings, scale measurements, parasite identification, otolith (earbone) analysis, and DNA identification, many of which can provide information on factors such as fish age and habitats traversed. Biological tags are used without prior capture of the fish, thus eliminating any potential effects of capture and handling (for reviews see [42]–[44]). Methods such as otolith sampling necessitate that the fish be killed prior to sampling, while other methods can be performed non-lethally. Although methods such as parasite and DNA identification may not be considered ‘tagging’ in a classical sense, such methods have been used to provide detailed information concerning the origin and movement patterns of the fish.

## Methods

Literature searches were carried out using two commercial academic search engines, ISI Web of Knowledge and Aquatic Sciences and Fisheries Abstracts, with a focus on peer-reviewed journal articles published in the English language as early as 1900 and extending to September 2011. We used combinations of key terms to focus search results on literature that used tagging as a method to study movement, behaviour, or survival in marine ecosystems of anadromous salmon within the genera *Oncorhynchus* and *Salmo*. Specifically, we focused on research of anadromous forms of Pacific salmon (pink, sockeye, Chinook, coho, chum, amago, masu), Atlantic salmon, as well as anadromous brown and sea trout, steelhead, and cutthroat trout (see **Appendix S1** for exact Boolean search terms).

Search results from both academic search engines were pooled and duplicates removed. All abstracts from resulting papers in the search databases were read in order to eliminate any papers that did not meet the criteria for inclusion in the literature review; the study had to involve some form of tagging of free-living salmonids (i.e. salmonids released into the natural environment) and results had to include information on behaviour or survival in the marine environment. A descriptive review was performed on the papers meeting our criteria.

For the descriptive review, a spreadsheet was first constructed with predetermined variables to be queried of each paper. The variables were chosen as a means to address the author's objectives, methods, and results. Examples of variables that were queried of papers include the year of study, author's motivation (i.e. basic biology, conservation, enhancement, fisheries management), geographic location, fish natal origin, species, life stages, tag types, author's inferred variables from tags (i.e. swim speed, travel behaviour, location, survival), handling/tagging effects (i.e. measured, acknowledged, not mentioned), tag loss/failure (i.e. measured, acknowledged, not mentioned), tag detection efficiency (i.e. measured, acknowledged, not mentioned), hatchery/farmed vs. wild fish, environmental variables tested, and physiological variables tested. Although we limited our descriptive review to peer-reviewed articles from our directed searches, information from relevant government and non-government agency reports were incorporated into the review where appropriate, but not into the numerical results.

## Results and Discussion

### General observations

**Figure 1:**
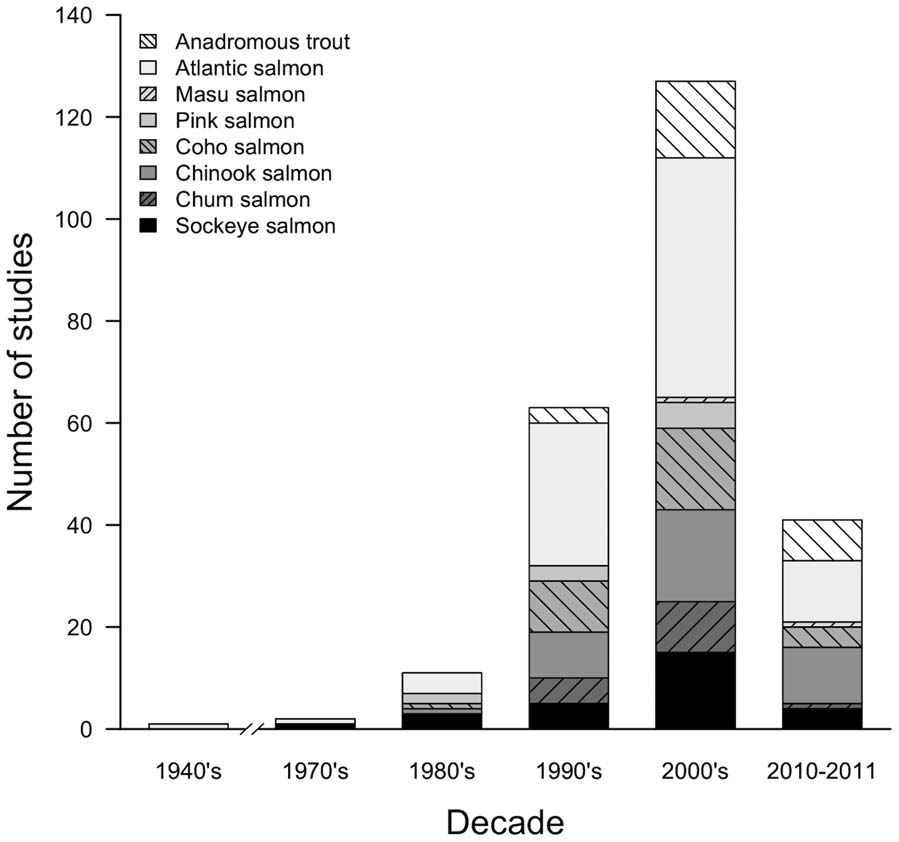
Number of study of particular species by publication decade. Total number of studies (n = 245) exceeds that of reviewed papers (n = 207) because many studies investigated more than one species. Steelhead, cutthroat, brown and sea trout were combined into “Anadromous trout”.

We identified 207 peer-reviewed articles (**Appendix S2**) published in the English language that met our criteria of using tagging in free-living fish to address anadromous salmonid behaviour or survival in the marine environments. The earliest publication resulting from our literature review appeared in 1940 [45]. As expected, the number of publications continuously increased since then (**Figs. 1** **,** **2**), reflecting an increasing use of tagging for gathering information on salmonids in marine environments. The main motivation for research was primarily the pursuit of basic biological information (75.4%; n = 156), followed by fisheries management (30.4%; n = 63), achievement of broad conservation goals (23.2%; n = 48), development or testing of tagging methodologies (14.5%; n = 30), assessment of salmon enhancement (14.5%; n = 30), assessment of habitat degradation (5.3%; n = 11), and climate change (2.9%; n = 6). Given the widespread recognition of the impacts that global warming is having and will probably continue to have on aquatic systems [46], it was surprising that climate change was the least identified motivation of any of the research we reviewed.

**Figure 2:**
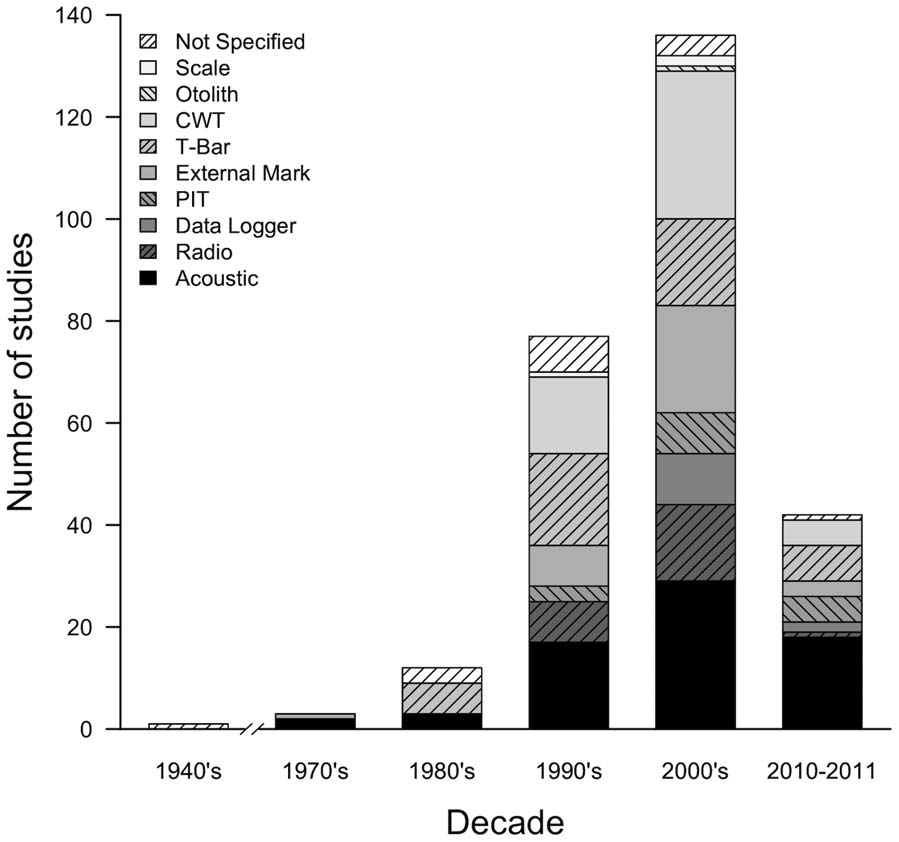
Number of use of specific tag types by publication decade. Total number of tag use (n = 271) exceeds that of reviewed papers (n = 207) because many studies used more than one tag type. The category “T-Bar” includes carlin, cinch, spaghetti, Floy, and Petersen disk tags. The plot does not include data from a paper published in 1940 because the tag type used was not specified by the author.

Irrespective of fish species or study origin, the majority of research in which geographic location was specifically defined (n = 207) was performed in the northeast Pacific Ocean (45.9%; n = 95) and the northeast Atlantic Ocean (35.7%; n = 74). Other locations included the northwest Atlantic Ocean (9.2%; n = 17), northwest Pacific Ocean (6.3%; n = 13), Bering Sea (3.9%; n = 8), and southern Pacific Ocean near New Zealand (1.9%; n = 4). Out of 206 studies that defined fish natal origin, fish stocks from Norway/Finland (24.6%; n = 46) and British Columbia/Puget Sound (24.1%; n = 55) have been the most studied, followed by the continental U.S. west coast (19.3%; n = 44), British Isles (9.2%; n = 21), eastern Canada/U.S. (8.8%; n = 20), Japan/Russia (6.6%; n = 15), Alaska (5.7%; n = 13) and New Zealand (1.8%; n = 4). Furthermore, the majority of studies examined fish of hatchery origin (37.7%; n = 60) compared to wild origin (12.6%; n = 20), ranched (sea cage) origin (4.7%; n = 7), a combination of wild and hatchery origin (30.0%; n = 47), or a combination of ranched and hatchery origin (3.1%; n = 5). Out of the total occurrences of species within the research [i.e. (n = 245) because some studies examined more than one species], Pacific salmon were the most frequently studied (69.1%; n = 143), whereas Atlantic salmon and anadromous trout comprised 45% (n = 93) and 4.4% (n = 9) of studies, respectively (**Fig. 1**). Within the Pacific salmonids, Chinook was the most studied (18.8%; n = 39), followed by coho (15.0%; n = 31), sockeye (13.5%; n = 28), steelhead (8.2%; n = 17), chum (7.7%; n = 16), pink (4.8%; n = 10), and masu (1.0%; n = 2) salmon. Overall, these results indicate very skewed distributions of research in terms of geographic location, species, and stock origins.

Various forms of tag technologies have been employed throughout the last half-century. Passive tag use has increased in recent decades, and out of the total number occurrences of tags [i.e. (n = 255) because some studies use more than one tag type], this was the most common tagging approach that we identified (57.3%; n = 146) (**Fig. 2**). In regard to the total number of occurrences of tags in the literature (n = 255), acoustic tags were the single most dominant tagging method (27.0%; n = 69), followed by CWTs (19.2%; n = 49), external visual tags (t-bar anchor) (19.0%; n = 48), external markings (12.9%; n = 33), radio tags (9.4%; n = 24), PIT tags (6.3%; n = 16), data loggers (4.7%; n = 12), and various forms of biological tags (e.g. otoliths, parasites, scales) (1.6%; n = 4). When external markings were used (n = 33) they were primarily combined with another form of tagging (75.8%; n = 25). When acoustic transmitters were used (n = 102), they were applied primarily to study juveniles (**Tables 1** **,** **2**). In contrast, when data loggers were used (n = 13), they were applied primarily to study adults in the open-ocean during or prior to their spawning migration to freshwater (**Tables 1** **,** **2**), likely reflecting tag size, and efforts to maximise tag retrievals by relocating fish once they arrive at spawning grounds. Biological tags are relatively new techniques and were used in only four (1.6%) studies. The low number of studies using biological tags may have been an artefact of the literature search terms being too narrow to locate more of these studies.

**Table 1:** Frequency (% within parentheses) of use of different tag types to study the life stages of anadromous salmonids in the marine environment for studies focusing on survival (i.e. those focusing on only survival and both on survival and behaviour).

	Tag type							
Life stage	Acoustic	Radio	Data Logger	PIT	CWT	External	Biological	Row Total
Out-migration (juveniles)	23 (63.9/42.6)	3 (20/5.6)	0 (0/0)	7 (53.8/13)	10 (25.6/18.5)	11 (19.3/20.4)	0 (0/0)	54 (NA/100)
Out-migratin (juveniles) to open ocean	2 (5.6/20)	0 (0/0)	0 (0/0)	0 (0/0)	2 (5.1/20)	6 (10.5/60)	0 (0/0)	10 (NA/100)
Out-migration (kelts)	3 (8.3/42.9)	0 (0/0)	1 (100/14.3)	1 (7.7/14.3)	0 (0/0)	2 (3.5/28.6)	0 (0/0)	7 (NA/100)
Return migration	4 (11.1/15.4)	11 (73.3/42.3)	0 (0/0)	0 (0/0)	2 (5.1/7.7)	9 (15.8/34.6)	0 (0/0)	26 (NA/100)
Open-ocean	0 (0/0)	0 (0/0)	0 (0/0)	0 (0/0)	1 (2.6/33.3)	2 (3.5/66.7)	0 (0/0)	3 (NA/100)
Open-ocean to return migration	1 (2.8/100)	0 (0/0)	0 (0/0)	0 (0/0)	0 (0/0)	0 (0/0)	0 (0/0)	1 (NA/100)
Entire life cycle (juvenile-return adult)	3 (8.3/4.9)	1 (6.7/1.6)	0 (0/0)	5 (38.5/8.2)	24 (61.5/39.3)	27 (47.4/44.3)	1 (100/1.6)	61 (NA/100)
Column Total	36 (100/NA)	15 (100/NA)	1 (100/NA)	13 (100/NA)	39 (100/NA)	57 (100/NA)	1 (100/NA)	162 (100/100)

The first % value within parentheses shows the relative frequency of use of a given tag type across life stages. The second % value shows the relative frequency of use of different tag types to study a particular life stage. The total frequency of tag use (n = 342) exceeds that of reviewed papers (n = 207) because many studies encompassed more than one life stage. The category “Biological” includes otoliths and scales, whereas the category “External” includes Carlin, cinch, Floy, Petersen disk tags and external markings.

**Table 2:** Frequency (% within parentheses) of use of different tag types to study the life stages of anadromous salmonids in the marine environment for studies focusing on behaviour (i.e. those focusing on only behaviour and both on behaviour and survival).

	Tag type							
Life stage	Acoustic	Radio	Data Logger	PIT	CWT	External	Biological	Row Total
Out-migration (juveniles)	36 (54.5/60)	3 (13.6/5)	1 (8.3/1.7)	6 (66.7/10)	4 (21.1/6.7)	9 (18.8/15)	1 (25/1.7)	60 (NA/100)
Out-migratin (juveniles) to open ocean	2 (3/20)	0 (0/0)	0 (0/0)	0 (0/0)	0 (0/0)	8 (16.7/80)	0 (0/0)	10 (NA/100)
Out-migration (kelts)	4 (6.1/40)	0 (0/0)	2 (16.7/20)	1 (11.1/10)	0 (0/0)	3 (6.3/30)	0 (0/0)	10 (NA/100)
Return migration	16 (24.2/32)	17 (77.3/34)	3 (25/6)	0 (0/0)	1 (5.3/2)	13 (27.1/26)	0 (0/0)	50 (NA/100)
Open-ocean	3 (4.5/27.3)	0 (0/0)	2 (16.7/18.2)	0 (0/0)	4 (21.1/36.4)	1 (2.1/9.1)	1 (25/9.1)	11 (NA/100)
Open-ocean to return migration	1 (1.5/25)	0 (0/0)	3 (25/75)	0 (0/0)	0 (0/0)	0 (0/0)	0 (0/0)	4 (NA/100)
Entire life cycle (juvenile-return adult)	4 (6.1/11.4)	2 (9.1/5.7)	1 (8.3/2.9)	2 (22.2/5.7)	10 (52.6/28.6)	14 (29.2/40)	2 (50/5.7)	35 (NA/100)
Column Total	66 (100/NA)	22 (100/NA)	12 (100/NA)	9 (100/NA)	19 (100/NA)	48 (100/NA)	4 (100/NA)	180 (100/100)

The first % value within parentheses shows the relative frequency of use of a given tag type across life stages. The second % value shows the relative frequency of use of different tag types to study a particular life stage. The total frequency of tag use (n = 342) exceeds that of reviewed papers (n = 207) because many studies encompassed more than one life stage. The category “Biological” includes otoliths and scales, whereas the category “External” includes Carlin, cinch, Floy, Petersen disk tags and external markings.

Nearly 40% of studies examined multiple life stages (36.2%; n = 75), whereas the majority only examined a single life stage (63.8%; n = 132). External tags and CWTs were used most frequently in studies that examined multiple life stages beginning at the juvenile stage (**Tables 3** **,** **4**), likely because they can be applied to large numbers of juvenile fish at a low cost, there is a publicly available database of CWT data [47], and because early marine juvenile survival is thought to be important when considering lifetime fitness. Acoustic transmitters were employed most frequently when the research objectives were to examine just one life stage (**Tables 3** **,** **4**), an issue largely related to limited transmitter battery life.

**Table 3:** Frequency (% within parentheses) of use of different tag types to study single and multiple life stages of anadromous salmonids in the marine environment for studies focusing on survival (i.e. those focusing on only survival and both on survival and behaviour).

	Tag type							
Type of study	Acoustic	Radio	Data Logger	PIT	CWT	External	Biological	Row Total
Single stage	33 (91.7/37.9)	14 (93.3/16.1)	1 (100/1.1)	8 (61.5/9.2)	10 (25.6/11.5)	21 (36.8/24.1)	0 (0/0)	87 (NA/100)
Multiple stages	3 (8.3/4)	1 (6.7/1.3)	0 (0/0)	5 (38.5/6.7)	29 (74.4/38.7)	36 (63.2/48)	1 (100/1.3)	75 (NA/100)
Column Total	36 (100/NA)	15 (100/NA)	1 (100/NA)	13 (100/NA)	39 (100/NA)	57 (100/NA)	1 (100/NA)	162 (100/100)

The first % value within parentheses shows the relative frequency of use of a given tag type across type of study. The second % value shows the relative frequency of use of different tag types to study one or multiple life stages. The total frequency of tag use (n = 340) exceeds that of reviewed papers (n = 207) because some studies used more than one type of tag. The category “Biological” includes otoliths and scales, whereas the category “External” includes carlin, cinch, Floy and Petersen disk tags and external markings.

**Table 4:** Frequency (% within parentheses) of use of different tag types to study single and multiple life stages of anadromous salmonids in the marine environment for studies focusing on behaviour (i.e. those focusing on only behaviour and both on behaviour and survival).

	Tag type							
Type of study	Acoustic	Radio	Data Logger	PIT	CWT	External	Biological	Row Total
Single stage	61 (93.8/47.7)	19 (90.5/14.8)	8 (66.7/6.3)	7 (77.8/5.5)	7 (36.8/5.5)	24 (50/18.8)	2 (50/1.6)	128 (NA/100)
Multiple stages	4 (6.2/8)	2 (9.5/4)	4 (33.3/8)	2 (22.2/4)	12 (63.2/24)	24 (50/48)	2 (50/4)	50 (NA/100)
Column Total	65 (100/NA)	21 (100/NA)	12 (100/NA)	9 (100/NA)	19 (100/NA)	48 (100/NA)	4 (100/NA)	178 (100/100)

The first % value within parentheses shows the relative frequency of use of a given tag type across type of study. The second % value shows the relative frequency of use of different tag types to study one or multiple life stages. The total frequency of tag use (n = 340) exceeds that of reviewed papers (n = 207) because some studies used more than one type of tag. The category “Biological” includes otoliths and scales, whereas the category “External” includes carlin, cinch, Floy and Petersen disk tags and external markings.

The most frequent variable authors inferred from tagging studies was survival (59.0%; n = 122), various travel behaviours (e.g. holding, vertical migrations) (44.0%; n = 91), assessments of fish position or location (37.2%; n = 77), swim speed (26.6%; n = 55), migration route (23.7%; n = 49) and origin (9.7%; n = 20). A large proportion of the studies did not directly assess potential mechanisms influencing survival or behaviour; less than half of the studies (45.0%; n = 93) reported on linking environmental variables to tagging results, and even fewer (13.5%; n = 28) looked for associations between individual physiology and tagging results. Temperature was the most common environmental variable found to be associated with behaviour (17.9%; n = 27) and survival (9.1%; n = 6) (**Table 5**). Among physiological variables the author's tested, energetic state of the fish was most commonly associated with behaviour (2.0%; n = 3), whereas osmoregulatory state of the fish was most commonly associated with survival (4.5%; n = 3) (**Table 5**). Other variables commonly found to be associated with behaviour or survival included fish size and stock effects (i.e. populations, wild versus hatchery) (**Table 5**). However, this does not necessarily mean that these particular variables are the most important in affecting salmonid behaviour or survival, as the variables were not equally tested for among studies.

**Table 5:** Number and frequency (% within parentheses) of a variable being found significant out of the total number of significant findings for behaviour (n = 151) or survival (n = 66).

		Study focus	
Category	Variable	Behaviour	Survival
**Environmental**	Temperature	27 (17.9)	6 (9.1)
	Depth	16 (10.6)	1 (1.5)
	Diel Effects	16 (10.6)	0 (0)
	Tide	15 (9.9)	0 (0)
	Current	8 (5.3)	0 (0)
	Salinity	7 (4.6)	2 (3)
	Productivity	2 (1.3)	3 (4.5)
	River Discharge	4 (2.6)	3 (4.5)
**Physiological**	Reproductive State	2 (1.3)	2 (3)
	Stress Hormones	0 (0)	1 (1.5)
	Ionoregulatory State	0 (0)	3 (4.5)
	Energetic Status	3 (2)	1 (1.5)
**Other**	Fish Size	16 (10.6)	15 (22.7)
	Stock	16 (10.6)	14 (21.2)
	Sex	2 (1.3)	1 (1.5)
	Release Date	4 (2.6)	4 (6.1)
	Release Location	2 (1.3)	3 (4.5)
	Trophic Effects	5 (3.3)	1 (1.5)
	Fisheries	1 (0.7)	4 (6.1)
	Predation	5 (3.3)	2 (3)
**Total**		151 (100)	66 (100)

Note that the table is based on studies focusing solely on behaviour or survival, but not both.

### Life-stage specific observations

#### Juvenile salmon and Atlantic salmon kelts

Most tagging studies focused on the juvenile portion of the salmon life cycle (65.7%; n = 136), likely because this life stage exhibits high and variable mortality rates, as well as a result of the ease of capture of fish, relatively high abundance, proximity to research institutions during outmigration (near river mouths and urban areas), and availability from hatchery programs. In contrast, research on the kelt life stage of Atlantic salmon was the least common focus, comprising only 5.0% (n = 6) of studies on iteroparous species (n = 119). Although less studied than juvenile out-migrations, kelt out-migration behaviour and survival patterns mirrored that of outmigrating smolts [48] so will be discussed in combination.

Juvenile pink, sockeye, steelhead, Atlantic salmon and Atlantic salmon kelts tend to move actively and rapidly through coastal (continental shelf) waters during out-migration to the ocean [48]–[54]. Chum, coho and Chinook tend to migrate at a much slower rate and can remain in coastal waters for longer periods of time [49], [50], [55], [56]. Apart from differences between species, movement rates through estuarine and coastal environments vary between population, fish origin (e.g. hatchery vs. wild; [49], [57], [58]) and body size [55], [57].

Juvenile salmon in coastal waters tend to migrate during ebb tides and at night [52], [53], [59], swimming actively within tides [60]–[62]. While estimates of swimming speed show some variability (e.g. from 0.53 body lengths per second (bl s^−1^) [58] up to 4 bl s^−1^ [52]), an average routine rate of 1 bl s^−1^ is common [63]. Laboratory swimming respirometry studies have found that a speed of 1 bl s^−1^ is associated with a minimum gross cost of transport [64]. Juveniles and kelts often exhibit clear diel vertical and horizontal movement patterns. Nocturnal migration tends to be more rapid than movement during the day [53], [59]. Swimming depth during the day tends to be quite shallow, within 1–3 m of the ocean surface, and even less (<0.5 m) during the night [65]. Changes in swimming depth and migration speed may be strongly related to temperature and salinity [66], or light conditions [65], the latter perhaps being a strategy related to predator avoidance [67]. Indeed, vertical movement trends may be closely linked to the feeding patterns of avian predators, resulting in movement downward in the water column during daylight hours [67].

Mortality during the juvenile out-migration stage is higher than during other marine life history stages, even when compared to the lengthy adult open-ocean stage [68]. Using acoustic telemetry, mortality of juveniles departing coastal waters has been shown to be very high [50], [58], although recent research has shown that juvenile mortality in the open ocean may be even higher [50]. Estimates of survival for early ocean migrating salmonids have been made for Atlantic salmon [58], [61], [69], [70], Chinook [50], [71], coho [50], [57], chum [72], sockeye [50], [73], steelhead [50], [68], [74]–[76] and anadromous brown trout [58]. Juvenile survival can be affected by a multitude of factors including predation [77]–[80], competition [81], parasites [82], [83], inability to osmoregulate [57], [80], [84], pollution [85], marine entry timing [58], [81], [86], [87], adverse ocean conditions (temperature, salinity, oxygen, pH, productivity) [88]–[90], dams [91], and smolt size [71], [88].

Furthermore, survival rates have been shown to differ between hatchery and wild fish. Survival estimates for wild fish tend to be higher than those for hatchery juveniles [19], [57], [68], [75], [76], [81], [92]–[95]. In one study, survival of wild steelhead smolts during migration away from inshore waters ranged from 18–39%, while hatchery smolt survival was 3% [76]. Trends such as this suggest a discrepancy in fitness between the two groups, possibly due to differences in physiology [57], behaviour [96], and size [88], [97]–[99].

#### Open-ocean

The open-ocean migration of salmon has been studied the least frequently, being the primary focus of only 8.7% (n = 18) of tagging studies. This is likely due to the difficulty of accessing fish within this environment, technological constraints, and associated financial costs. In fact, much of what we know about salmon migration in the open-ocean comes from early research by fisheries capture and the use of external tags. This type of research was performed by international organizations such as the North Atlantic Salmon Conservation Organization (NASCO) (Atlantic salmon), and the North Pacific Anadromous Fish Commission (NPAFC) (Pacific salmon), which provided some of the first scientific insights into the open-ocean behaviour and ecology of anadromous salmon at sea. This early research revealed that salmon populations are often highly mixed at sea. For example, Pacific salmonid stocks from Japan, Russia, Canada, and the United States utilize several of the same marine feeding grounds [100]–[105].

A small number of recent studies have utilized recovery of data loggers and manual tracking of fish tagged with acoustic transmitters to assess fine scale movements of salmon in the open-ocean. Salmon migrating in the open-ocean tend to swim at speeds of 1 bl s^−1^ on average [106]–[108], which is similar to average swim speeds observed in other life stages (see above). Vertical distribution in the water column varies diurnally, seasonally, and by species (e.g. Chinook dive below 50 m whereas most other species remain within the upper 20 m of the water column [107]), as determined by acoustic tracking of tagged individuals [106], [107], [109] and data logger recoveries [110]–[113]. Vertical migrations are most likely related to maximizing foraging efficiency [112], predator avoidance, and for navigational purposes [114].

There are several factors that are thought to influence salmon survival in the open-ocean, including migration routes, timing, food availability, predator levels, ocean conditions [115]–[119] and carry-over effects from earlier life stages [120]. However, relatively few tagging studies have estimated survival in the open-ocean and the limited results suggest that survival rates can vary considerably among species and populations, and the causes remain poorly understood. For example, to investigate trends in survival across the Northeast Pacific over a long time scale, a study using CWT data from coho salmon found that ocean survival of northern stocks (northern BC and Alaska) increased from the 1980s to 1990, whereas survival of southern stocks has been declining over the same time period [24]. This inverse-covariablity between northern and southern latitude salmon production has been similarly shown in other salmonid species [121], and is thought to be associated with changing ocean regimes [122], [123]. However, using CWT data, a more recent study found no significant inverse-covariability on interannual timescales between northern and southern stocks of coho salmon [124], which demonstrates our lack of understanding on the processes influencing population dynamics of salmonids in the open-ocean.

#### Return migration

Although they can travel thousands of kilometres in high seas, most maturing salmon have the ability to navigate back to natal freshwater streams upon reaching maturity. Nevertheless, straying behaviours (e.g. individuals spawning in non-natal waters) are present in several species [22], [125]–[129] and may represent an important evolutionary survival strategy. Even though some populations in certain watersheds (e.g. the Fraser River) have recently exhibited variable river entry timing [130], upriver spawning migrations by mature adults usually commence within the same week each year [131], [132]. Such predictability certainly facilitates the study of this life stage, which ranked second in our analysis (22.2%; n = 46).

Timing and location of arrival of salmon to the continental shelf from ocean feeding grounds is based on environmental factors in the ocean [56], [133]–[135] and physiological state of the fish [136], [137]. Swim speed for returning adults has been determined simplistically using manual tracking of individuals [107], [138], and by more sophisticated means using data loggers that directly measure swim speed [139]. Again it emerges that adult salmon are observed to routinely swim at average speeds around 1 bl s^−1^ [41], [107], [138]. Migration rates and timing are influenced by a range of environmental factors (reviewed in [140]), some of which are tides, currents, salinity levels and temperature [141]–[147]. As in the open-ocean, vertical position in the water column in coastal areas can vary among species and even within species between relatively short distances on continental shelves. For example, manually tracked sockeye salmon were observed to choose different depths when swimming in well mixed coastal waters versus stratified coastal waters, preferring deeper water when they encountered a stratified water column created by river discharge [138]. Vertical movements may be related to species preferring narrow ranges of temperature [148]. Several species continue to exhibit diel vertical movement patterns during this portion of their life [112], [138], [144], [149], which may be a behaviour used to conserve energy prior to river migration, avoid predators, prepare osmotically for freshwater entry, or aid in navigation [140], [141], [148].

Though only a few studies have focused on aspects of salmonid physiology, the role of physiological state as a key driver of return migration behaviour and survival is highlighted by a series of studies conducted on Fraser River sockeye salmon. Specifically, fish with advanced reproductive preparedness (e.g. elevated plasma concentrations of reproductive hormones, including testosterone, 11-ketotestosterone, and 17β-estradiol) migrated fastest coastally and entered the river earlier [137], [150], [151]. Marine survival was related to physiological stress such that fish with elevated plasma ion glucose and lactate levels perished in coastal waters before entering the river [150], [152], [153]. Survival was also lower in fish that were less physiologically prepared for freshwater entry (i.e. higher plasma chloride and total osmolality [154]). These studies provide examples of how telemetry can be combined with physiological measurements to address research questions.

### Knowledge gaps and future directions

#### Core knowledge

Our review identified several priority areas for research due to inadequate investigation to date. We believe these knowledge gaps constrain the current understanding of salmon in marine environments, and potentially limit the application of contemporary tagging technologies for management and conservation purposes. Below, we discuss each area and give recommendations to address these concerns wherever possible.

Globally, knowledge of the impact of climate change on salmon behaviour and survival in the marine environment is limited. Less than half of tagging studies analyzed in this review attempted to link abiotic factors such as temperature, salinity, oxygen, and productivity to salmon behaviour or survival (except see [13], [155]–[160]). Major climatic changes have already occurred [55], and shifts in ocean temperatures, salinity, oxygen concentration, pH, and prey abundance are expected to intensify [161], [162], with profound compounding effects on salmonid distribution and survival [5]. Tagging can be a powerful tool to increase our understanding of the impacts of environmental change on salmonids, particularly if studies are long-term and combined with effective environmental monitoring (e.g. through the use of data loggers). Furthermore, experimental studies that manipulate temperature or salinity can be combined with biopsy and telemetry techniques to further contribute to the knowledge base (e.g. [163]).

In addition, certain regions (e.g. Bering Sea, northwest Pacific Ocean, New Zealand), populations (e.g. those from Alaska, Japan/Russia, New Zealand), life stages (e.g. open-ocean, kelts) and species (e.g. pink, masu) are underrepresented in the literature. Most of the research we analyzed examined hatchery fish rather than wild fish, and relatively few tagging studies compared the two (except see [19], [49], [50], [57], [68], [75], [76], [81], [92]–[95], [164], [165]), despite known differences in behaviour and survival. For example, wild populations commonly display adaptive plasticity in migration timing due to environmental variation and as a means of avoiding interspecies competition [81], while hatchery raised fish are manually released according to a hatchery schedule [92]. Hatchery fish often have lower fitness and subsequent survival in natural environments than wild stocks [92]. This suggests that conclusions from tagging studies using hatchery fish should perhaps not be applied broadly to wild populations. Tagging studies among populations, as well as between hatchery and wild fish, could provide insights into key differences among such groups.

There are also limited data on full life cycle analyses, as very few studies assess more than one life stage at one time, a method that does not account for any cumulative effects throughout the life history. For instance, juvenile growth rates can affect fitness and survival in all remaining life stages, and successful development at sea may have cascading effects on subsequent reproductive maturation and spawning success. Tagging juveniles and assessing the entire life cycle while monitoring abiotic factors may provide powerful insights into which environmental effects have the greatest impact on lifetime fitness. Various technologies exist that could be implemented on a large scale relatively inexpensively, such as external visual tags or PIT tags, however, more expensive acoustic tags or data loggers could provide more detailed information on both biotic and abiotic factors.

Finally, we identified a definite lack of research on salmonid survival and mortality at sea. Although some research has looked at lifetime survival through tagging, these studies were unable to determine exactly where and why mortality occurs. Understanding lifetime survival rates is critical to understanding population viability, yet there is no conclusive data to date to suggest which life stage is associated with the highest mortality. This has made it challenging to relate environmental variables to mortality across life stages. While current technologies cannot yet provide precise estimates of location and cause of mortality, this may change in the near future. For example, to control the problem of limited battery life, acoustic transmitters have now been designed that can ‘turn off’ while salmon are at sea, and then power-up 2–3 years later upon return migration to freshwater where they can be tracked with acoustic arrays [73].

#### Tagging models, procedures and technologies

A common feature of studies designed to estimate survival from tagged animals in the wild is the potential for imperfect (i.e. <100%) encounter (i.e. detection or recapture of electronic and passive tags, respectively) probabilities. When researchers do not account for encounter probabilities that are <100%, survival estimates will be biased low, and erroneous interpretations of results can occur in cases where encounter probabilities vary among tagged fish belonging to different strata (e.g. sex) or assigned to different experimental treatments [166]. Capture-recapture models for open populations have been developed since the 1960's to deal explicitly with imperfect encounter probabilities in the estimation of survival and other demographic parameters from tagged animals [167]. However, despite the long-standing availability and continued development of capture-recapture models and specialized computer software for their implementation, only 20.9% (n = 23 out of 110) of the studies where capture-recapture models were applicable have accounted for imperfect encounter probabilities in the estimation of survival for anadromous salmonids. Indeed, in general there seems to be little appreciation and use of capture-recapture models in fisheries research [168]. Encounter probabilities have been measured for multiple species of salmon smolts using the Pacific Ocean Shelf Tracking (POST) array in coastal waters (reviewed in [50]).

A related class of models allows researchers to estimate survival from tag recoveries of harvested animals [169] or from both live encounters and tag recoveries [170]. Tag recovery, whether by commercial fisheries or by other means, was used in 63.5% (n = 129) of studies reporting how tag data were retrieved. The use of models based on tag-recovery data to estimate survival could be applied to these studies. An interesting application of models based on tag recovery is the possibility to separate fishing from natural mortality if an estimate of tag reporting probability is available [168]. Several experiments have been proposed to estimate the probability that tags are reported. For example, reporting probability can be estimated as the recovery of standard (i.e. no- or low-reward) tags relative to high-reward tags (assuming these are 100% reported if encountered) [171]; or by planting tags into fisheries catches and calculating the ratio between planted tags reported and the known number of tags that were planted [172].

Both capture-recapture and tag-recovery models are based on the assumption that tags are not lost or shed and, in the case of electronic tags, that they do not fail. If this assumption is violated, survival will be underestimated [167]. However, tag loss/shed was measured in only 7.7% (n = 16) of studies and was not even acknowledged in 69.0% (n = 143) of studies where it could possibly have occurred. Double-tagging individuals could minimize the impacts of tag loss/shed on survival estimates [173], an approach that was employed in 28.5% (n = 59) of the studies. Assuming that loss/shed of the two tags are independent, information on the number of fish recaptured with one or both tags could be used to estimate the probability of tag loss/shed and then used to adjust survival estimates [173]. Alternatively, multistate capture-recapture models could be used to jointly estimate survival and tag/shed loss [174]. When looking exclusively at studies using electronic tags (n = 69), 11.6% (n = 8) measured tag failure. These measures are important as they allow researchers to construct time to failure curves for the electronic tags. This information, along with fish detection times, can be incorporated into the likelihood function of a capture-recapture model to account for tag failure into estimates of survival [175], [176]. A similar approach could also be used to account for the loss/shed of passive tags into survival estimates [175].

Another important assumption of capture-recapture and tag-recovery models is that tagging does not affect survival; otherwise survival estimates will be biased low [167]. Capture methods [177], tag types [178], tagging methods (e.g. external attachment, surgical application, gastric insertion, injection) [179], [180], the use of anesthetics, handling time, tag size and release technique (e.g. recovery period) can all impact survival of the tagged fish. Capture-recapture models can be modified to account for short-term tagging effects on survival of newly tagged individuals [169]. In fact, tagging effects are not only issues in studies of survival but also of movement and behaviour [179], [181]. However, only 10.6% (n = 22) of studies assessed tagging/handling effects, and an acknowledgment of potential tagging/handling effects was made in only 33.8% (n = 70) of studies. Tag size is a major limitation in salmon research, especially in studies of juvenile fish [182], and very few of the studies we reviewed assessed survival costs or tag burdens on juveniles (except see [57], [76], [180], [183]–[186]). While there have been a number of studies performed under laboratory settings to assess tag effects to supplement field studies or to model tag limits for certain species [63], [180], [187]–[189], few studies conducted these trials under field conditions (except see [50], [188], [190]).

Remarkable advancements have been made in the field of fish tagging throughout the last few decades. Movement towards electronic rather than passive tags has enabled researchers to more thoroughly investigate the movement and survival patterns of individual salmonids in the marine environment. Nevertheless, the historic (and ongoing) studies that utilized passive tags (primarily CWT, and/or adipose fin clip) remain some of the most enlightening due to their large sample sizes across multiple years (cost effectively), and their applicability to very small juveniles. Clearly, this is an area where current electronic tagging technologies require further advancement to minimize costs, decrease tag sizes, and thus allow long-term studies to be conducted with an aim to more comprehensively examine the interannual variability in salmonid biology.

Indeed, some manufacturers have concentrated on miniaturizing electronic tags such that they are of use in very small fish, such as the recently developed JSAT tags [182]. At present, many of these miniaturized tags emit an acoustic or radio signal such that the fish can be detected when they swim within range of particular receivers. Owing to these tags, an excellent database is accumulating regarding the early marine phase of the lifecycle of salmon smolts, including aspects of behaviour and survival [50], [57], [68], [72], [73], [75], [76]. A limitation of these studies is that fish must be presumed dead if they are not detected on subsequent receivers following their detection on a prior receiver. This results in areas between receivers where many fish may have disappeared for reasons that cannot be ascertained with current technologies and infrastructure.

Though not frequently used yet, multi-sensor tags are one future development that holds considerable promise as they allow detailed insight into the behaviour (e.g. acceleration, tail beat frequency, dive patterns) and physiology (e.g. heart rate, blood oxygen status) of individual fish in the natural environment [35]–[39], [41]. While many adult salmon can accommodate certain multi-sensor tags, miniaturization of the tags to the point where they can be used in smolts is some distance into the future. Multi-sensor tags are typically archival due to the inherent difficulties of transmitting data from multiple sensors to a receiver during the transient period when the fish is in range. This limitation is guiding engineering research to develop archival tags that transmit stored data intermittently to receivers whenever the fish is in range. The transmission will continue where it left off once the fish is in range of a subsequent receiver. An exciting prospect is that other animals may ultimately act as ‘receivers’. That is, large animals (e.g. sharks, whales) that are capable of carrying a PSAT, for example, could receive data from nearby smaller animals (e.g. juvenile and adult salmon) and transmit the data both from themselves and from the smaller animals to satellite receivers. Termed ‘business card’ tags, these technologies promise exciting avenues for salmon research in the future [191].

#### Conclusions

Tagging and telemetry are tools that have the potential to integrate research and researchers across disciplines to advance our knowledge of salmonid behaviour, physiology and survival. By combining passive and newly emerging electronic and biological tagging approaches, incorporating environmental, physiological and behavioural observations into tagging studies, and utilizing broad-scale telemetry arrays and curtains (e.g. Pacific Ocean Shelf Tracking Project – POST, Ocean Tracking Network – OTN, Tagging of Pelagic Predators – TOPP), multi-life stage and multi-trophic level investigations are within reach (see [192]). Finally, international collaboration, as is occurring in projects such as OTN and TOPP, will greatly benefit salmonid research in the marine environment.

## Supporting Information

Appendix S1 **Boolean search terms used in Web of Science and Aquatic Science and Fisheries Abstracts academic search engines to locate peer-reviewed literature.**
(DOC)

Appendix S2 **References for peer-reviewed articles that were incorporated into descriptive review (i.e. numerical results).**
(DOC)
