# Supplementary material for: A Synthesis of Tagging Studies Examining the Behaviour and Survival of Anadromous Salmonids in Marine Environments
Source: PLoS One. 2012 Mar 14;7(3):e31311. doi: 10.1371/journal.pone.0031311 (PMC3303779; doi:10.1371/journal.pone.0031311)
Supplement: Appendix S1 — Boolean search terms used in Web of Science and Aquatic Science and Fisheries Abstracts academic search engines to locate peer-reviewed literature. (DOC) [file pone.0031311.s001.doc]

**Appendix S1**

**Web of Science Search Terms:**

TS=(**ocean*** or **marine*** or **coast*** or **estuar***) AND TI=(**salmon*** or **oncorhynchus** or **salmo** or **steelhead** or **cutthroat**) AND TS=(**mortality** or **survival** or **dispersal** or **behavio$r** or **migrat*** or **movement** or **physiolog***) AND TS=(**telemetry** or **tag*** or **track*** or **transmit*** or "**data** **logger**") NOT TS=(**salmonella*** or **paleo*** or **shark***) NOT TI=(**cod** or **herring** or **mullet** or **oyster*** or **mussel*** or **bass** or **sunfish*** or **charr** or **shrimp** or **eel** or **gilthead** or **bream** or **turbot** or **alosa** or **trutta**) NOT SO=(**Food** **Chemistry** or **Aquaculture** **Nutrition** or **Aquaculture** **Research** or **Aquaculture** **Engineering**)

**ASFA Search Terms:**

KW=(**salmon*** or **oncorhynchus** or **salmo**) and KW=(**ocean*** or **marine*** or **coast*** or **estuar***) and KW=(**mortality** or **survival** or **dispersal** or **behavio**$**r** or **migrat*** or **movement** or **telemetry** or **tag*** or **track*** or **physiolog*** or **temperature** or **salinity** or **fisher*** or **climate** or **predat***) and not TI=(**cod** or **mykiss** or **herring** or **mullet** or **oyster*** or **mussel*** or **bass** or **sunfish*** or **charr** or **shrimp** or **eel** or **gilthead** or **bream** or **turbot** or **alosa** or **trutta**) and not JN=(**food chemistry** or **veterinary microbiology** or **nutrition** or **shellfish** or **ornithology**) and not KW=(**salmonella*** or **shark*** or **paleo***)
