# Supplementary material for: A Synthesis of Tagging Studies Examining the Behaviour and Survival of Anadromous Salmonids in Marine Environments
Source: PLoS One. 2012 Mar 14;7(3):e31311. doi: 10.1371/journal.pone.0031311 (PMC3303779; doi:10.1371/journal.pone.0031311)
Supplement: Appendix S2 — References for peer-reviewed articles that were incorporated into descriptive review (i.e. numerical results). (DOC) [file pone.0031311.s002.doc]

**Appendix S2**

Aarestrup, K., Jepsen, N., Rasmussen, G., Okland, F., Thorstad, E.B., Holdensgaard, G. (2000) Prespawning migratory behaviour and spawning success of sea-ranched Atlantic salmon, *Salmo salar L*., in the River Gudenaa, Denmark. *Fish. Manage. Ecol.* **7**, 387-400.

Ando, D., Miyamoto, M., Kasugai, K., Miyakoshi, Y., Nagata, M. (2005) Seasonal distribution of yearling masu salmon released from the Sea of Japan side of southwestern Hokkaido, Japan. *N. Am. J. Fish. Manage.* **25**, 22-37.

Aprahamian, M.W., Jones, G.O. (1997) The seaward movement of Atlantic salmon smolts in the Usk estuary, Wales, as inferred from power station catches. *J. Fish Biol.* **50**, 442-444.

Aprahamian, M.W., Jones, G.O., Gough, P.J. (1998) Movement of adult Atlantic salmon in the Usk estuary, Wales. *J. Fish Biol.* **53**, 221-225.

Azumaya, T., Ishida, Y. (2005) Mechanism of body cavity temperature regulation of chum salmon (*Oncorhynchus keta*) during homing migration in the North Pacific Ocean. *Fish Oceanogr* **14**, 81-96.

Bailey, R.E., Irvine, J.R., Dalziel, F.C., Nelson, T.C. (1998) Evaluations of visible implant fluorescent tags for marking coho salmon smolts. *N. Am. J. Fish. Manage.* **18**, 191-196.

Balfry, S., Welch, D.W., Atkinson, J., Lill, A., Vincent, S. (2011) The effect of hatchery release strategy on marine migratory behaviour and apparent survival of Seymour River steelhead smolts (*Oncorhynchus mykiss*). *Plos One* **6**, e14779. doi:14710.11371/journal.pone.0014779.

Beamish, R.J., Sweeting, R.M., Lange, K.L., Neville, C.M. (2008) Changes in the population ecology of hatchery and wild coho salmon in the Strait of Georgia. *Trans. Am. Fish. Soc.* **137**, 503-520.

Belding, D.L. (1940) Migration of the Atlantic salmon (*Salmo salar*) in the Gulf of St. Lawrence as determined by tagging experiments. *Trans. Am. Fish. Soc.* **69**, 290-295.

Birtwell, I.K., Fink, R., Brand, D., Alexander, R., McAllister, C.D. (1999) Survival of pink salmon (*Oncorhynchus gorbuscha*) fry to adulthood following a 10-day exposure to the aromatic hydrocarbon water-soluble fraction of crude oil and release to the Pacific Ocean. *Can. J. Fish. Aquat. Sci.* **56**, 2087-2098.

Bond, M.H., Hanson, C.V., Baertsch, R., Hayes, S.A., MacFarlane, R.B. (2007) A new low-cost instream antenna system for tracking passive integrated transponder (PIT)-tagged fish in small streams. *Trans. Am. Fish. Soc.* **136**, 562-566.

Bond, M.H., Hayes, S.A., Hanson, C.V., MacFarlane, R.B. (2008) Marine survival of steelhead (*Oncorhynchus mykiss*) enhanced by a seasonally closed estuary. *Can. J. Fish. Aquat. Sci.* **65**, 2242-2252.

Booker, D.J., Wells, N.C., Smith, I.P. (2008) Modelling the trajectories of migrating Atlantic salmon (*Salmo salar*). *Can. J. Fish. Aquat. Sci.* **65**, 352-361.

Brawn, V.M. (1982) Behavior of Atlantic salmon (*Salmo salar*) during suspended migration in an estuary, Sheet Harbor, Nova-Scotia, observed visually and by ultrasonic tracking. *Can. J. Fish. Aquat. Sci.* **39**, 248-256.

Buchanan, R.A., Skalski, J.R. (2007) A migratory life-cycle release-recapture model for salmonid PIT-tag investigations. *Journal of Agricultural Biological and Environmental Statistics* **12**, 325-345.

Buchanan, R.A., Skalski, J.R., Giorgi, A.E. (2010) Evaluating surrogacy of hatchery releases for the performance of wild yearling Chinook salmon from the Snake River basin. *N. Am. J. Fish. Manage.* **30**, 1258-1269.

Bugert, R.M., Mendel, G.W. (1997) Adult returns of subyearling and yearling fall Chinook salmon released from a Snake River hatchery or transported downstream. *N. Am. J. Fish. Manage.* **17**, 638-651.

Buoro, M., Prevost, E., Gimenez, O. (2010) Investigating evolutionary trade-offs in wild populations of Atlantic salmon (*Salmo salar*): incorporating detection probabilities and individual heterogeneity. *Evolution* **64**, 2629-2642.

Candy, J.R., Beacham, T.D. (2000) Patterns of homing and straying in southern British Columbia coded-wire tagged Chinook salmon (*Oncorhynchus tshawytscha*) populations. *Fish. Res.* **47**, 41-56.

Candy, J.R., Quinn, T.P. (1999) Behavior of adult Chinook salmon (*Oncorhynchus tshawytscha*) in British Columbia coastal waters determined from ultrasonic telemetry. *Can J Zool* **77**, 1161-1169.

Chamberlin, J.W., Kagley, A.N., Fresh, K.L., Quinn, T.P. (2011) Movements of yearling Chinook salmon during the first summer in marine waters of Hood Canal, Washington. *Trans. Am. Fish. Soc.* **140**, 429-439.

Chittenden, C.M., Jensen, J.L.A., Ewart, D., Anderson, S., Balfry, S., Downey, E., Eaves, A., Saksida, S., Smith, B., Vincent, S., Welch, D., McKinley, R.S. (2010a) Recent salmon declines: a result of lost feeding opportunities due to bad timing? *Plos One* **5**, e1242310.1241371/journal.pone.0012423.

Chittenden, C.M., Melnychuk, M.C., Welch, D.W., McKinley, R.S. (2010b) An investigation into the poor survival of an endagered coho salmon population. *Plos One* **5**, e10869. doi:10810.11371/journal.pone.0010869.

Chittenden, C.M., Rikardsen, A.H., Skilbrei, O.T., Davidsen, J.G., Halttunen, E., Skardhamar, J., McKinley, R.S. (2011) An effective method for the recapture of escaped farmed salmon. *Aquaculture Environment Interactions* **1**, 215-224.

Chittenden, C.M., Sura, S., Butterworth, K.G., Cubitt, K.F., Manel-La, N.P., Balfry, S., Okland, F., McKinley, R.S. (2008) Riverine, estuarine and marine migratory behaviour and physiology of wild and hatchery-reared coho salmon *Oncorhynchus kisutch* (Walbaum) smolts descending the Campbell River, BC, Canada. *J. Fish Biol.* **72**, 614-628.

Clemens, B.J., Clements, S.P., Karnowski, M.D., Jepsen, D.B., Gitelman, A.I., Schreck, C.B. (2009) Effects of transportation and other factors on survival estimates of juvenile salmonids in the unimpounded lower Columbia River. *Trans. Am. Fish. Soc.* **138**, 169-188.

Collis, K., Roby, D.D., Craig, D.P., Ryan, B.A., Ledgerwood, R.D. (2001) Colonial waterbird predation on juvenile salmonids tagged with passive integrated transponders in the Columbia river estuary: Vulnerability of different salmonid species, stocks, and rearing types. *Trans. Am. Fish. Soc.* **130**, 385-396.

Cooke, S.J., Crossin, G.T., Patterson, D.A., English, K.K., Hinch, S.G., Young, J.L., Alexander, R.F., Healey, M.C., Van der Kraak, G., Farrell, A.P. (2005) Coupling non-invasive physiological assessments with telemetry to understand inter-individual variation in behaviour and survivorship of sockeye salmon: development and validation of a technique. *J. Fish Biol.* **67**, 1342-1358.

Cooke, S.J., Donaldson, M.R., Hinch, S.G., Crossin, G.T., Patterson, D.A., Hanson, K.C., English, K.K., Shrimpton, J.M., Farrell, A.P. (2009) Is fishing selective for physiological and energetic characteristics in migratory adult sockeye salmon? *Evol. Appl.* **2**, 299-311.

Cooke, S.J., Hinch, S.G., Crossin, G.T., Patterson, D.A., English, K.K., Healey, M.C., Macdonald, J.S., Shrimpton, J.M., Young, J.L., Lister, A., Van Der Kraak, G., Farrell, A.P. (2008) Physiological correlates of coastal arrival and river entry timing in late summer Fraser River sockeye salmon (*Oncorhynchus nerka*). *Behav. Ecol.* **19**, 747-758.

Cooke, S.J., Hinch, S.G., Crossin, G.T., Patterson, D.A., English, K.K., Healey, M.C., Shrimpton, J.M., Van Der Kraak, G., Farrell, A.P. (2006a) Mechanistic basis of individual mortality in Pacific salmon during spawning migrations. *Ecology* **87**, 1575-1586.

Cooke, S.J., Hinch, S.G., Crossin, G.T., Patterson, D.A., English, K.K., Shrimpton, J.M., Van der Kraak, G., Farrell, A.P. (2006b) Physiology of individual late-run Fraser River sockeye salmon (*Oncorhynchus nerka*) sampled in the ocean correlates with fate during spawning migration. *Can. J. Fish. Aquat. Sci.* **63**, 1469-1480.

Coronado, C., Hilborn, R. (1998) Spatial and temporal factors affecting survival in coho salmon (*Oncorhynchus kisutch*) in the Pacific Northwest. *Can. J. Fish. Aquat. Sci.* **55**, 2067-2077.

Cotter, D., O'Donovan, V., O'Maoileidigh, N., Rogan, G., Roche, N., Wilkins, N.P. (2000) An evaluation of the use of triploid Atlantic salmon (*Salmo salar* L.) in minimising the impact of escaped farmed salmon on wild populations. *Aquaculture* **186**, 61-75.

Courtney, D.L., Mortensen, D.G., Orsi, J.A., Munk, K.M. (2000) Origin of juvenile Pacific salmon recovered from coastal southeastern Alaska identified by otolith thermal marks and coded wire tags. *Fish. Res.* **46**, 267-278.

Crossin, G.T., Hinch, S.G., Cooke, S.J., Cooperman, M.S., Patterson, D.A., Welch, D.W., Hanson, K.C., Olsson, I., English, K.K., Farrell, A.P. (2009a) Mechanisms influencing the timing and success of reproductive migration in a capital breeding semelparous fish species, the sockeye salmon. *Physiol. Biochem. Zool.* **82**, 635-652.

Crossin, G.T., Hinch, S.G., Cooke, S.J., Welch, D.W., Batten, S.D., Patterson, D.A., Van Der Kraak, G., Shrimpton, J.M., Farrell, A.P. (2007) Behaviour and physiology of sockeye salmon homing through coastal waters to a natal river. *Mar Biol* **152**, 905-918.

Crossin, G.T., Hinch, S.G., Welch, D.W., Cooke, S.J., Patterson, D.A., Hills, J.A., Zohar, Y., Klenke, U., Jacobs, M.C., Pon, L.B., Winchell, P.M., Farrell, A.P. (2009b) Physiological profiles of sockeye salmon in the Northeastern Pacific Ocean and the effects of exogenous GnRH and testosterone on rates of homeward migration. *Mar. Freshwat. Behav. Physiol.* **42**, 89-108.

Crozier, W.W., Kennedy, G.J.A. (1994) Marine exploitation of Atlantic salmon (*Salmo salar* L) from the River Bush, Northern-Ireland. *Fish. Res.* **19**, 141-155.

Crozier, W.W., Kennedy, G.J.A. (2002) Impact of tagging with coded wire tags on marine survival of wild Atlantic salmon (*Salmo salar* L.) migrating from the R. Bush, Northern Ireland. *Fish. Res.* **59**, 209-215.

Dat, C.G., Leblond, P.H., Thomson, K.A., Ingraham, W.J., Jr. (1995) Computer simulations of homeward-migrating Fraser River sockeye salmon: Is compass orientation a sufficient direction-finding mechanism in the north-east Pacific Ocean? *Fish Oceanogr* **4**, 209-216.

Davidsen, J.G., Manel-la, N.P., Okland, F., Diserud, O.H., Thorstad, E.B., Finstad, B., Sivertsgard, R., McKinley, R.S., Rikardsen, A.H. (2008) Changes in swimming depths of Atlantic salmon *Salmo salar* post-smolts relative to light intensity. *J. Fish Biol.* **73**, 1065-1074.

Davidsen, J.G., Rikardsen, A.H., Halttunen, E., Thorstad, E.B., Okland, F., Letcher, B.H., Skardhamar, J., Naesje, T.F. (2009) Migratory behaviour and survival rates of wild northern Atlantic salmon *Salmo salar* post-smolts: effects of environmental factors. *J. Fish Biol.* **75**, 1700-1718.

Dempson, J.B., Pepper, V.A., Furey, G., Bloom, M., Nicholls, T., Hoskins, G. (1999) Evaluation of an alternative strategy to enhance salmon populations: Cage rearing wild smolts from Conne River, Newfoundland. *ICES J. Mar. Sci.* **56**, 422-432.

Dempson, J.B., Robertson, M.J., Pennell, C.J., Furey, G., Bloom, M., Shears, M., Ollerhead, L.M.N., Clarke, K.D., Hinks, R., Robertson, G.J. (2011) Residency time, migration route and survival of Atlantic salmon *Salmo salar* smolts in a Canadian fjord. *J. Fish Biol.* **78**, 1976-1992.

Dieperink, C., Bak, B.D., Pedersen, L.F., Pedersen, M.I., Pedersen, S. (2002) Predation on Atlantic salmon and sea trout during their first days as postsmolts. *J. Fish Biol.* **61**, 848-852.

Duffy, E.J., Beauchamp, D.A. (2011) Rapid growth in the early marine period improves the marine survival of Chinook salmon (*Oncorhynchus tshawytscha*) in Puget Sound, Washington. *Can. J. Fish. Aquat. Sci.* **68**, 232-240.

English, K.K., Koski, W.R., Sliwinski, C., Blakley, A., Cass, A., Woodey, J.C. (2005) Migration timing and river survival of late-run Fraser River sockeye salmon estimated using radiotelemetry techniques. *Trans. Am. Fish. Soc.* **134**, 1342-1365.

Eriksson, T., Eriksson, L.O. (1991) Spawning migratory behavior of coastal-released Baltic salmon (*Salmo salar*) - effects on straying frequency and time of river ascent. *Aquaculture* **98**, 79-87.

Erkamo, E., Salminen, M., Salmi, J., Soederholm-Tana, L. (1994) Feeding, food quality, and nutritional state of salmon post-smolts in the Finnish coast of the Bothnian Sea. *Nord. J. Freshwat. Res.* **69**, no.

Erkinaro, J., Okland, F., Moen, K., Niemelae, E., Rahiala, M. (1999) Return migration of Atlantic salmon in the River Tana: the role of environmental factors. *J. Fish Biol.* **55**, 506-516.

Evans, A.F., Roby, D.D., Collis, K., Cramer, B.M., Sheggeby, J.A., Adrean, L.J., Battaglia, D.S., Lyons, D.E. (2011) Recovery of coded wire tags at a Caspian tern colony in San Francisco Bay: a technique to evaluate impacts of avian predation on juvenile salmonids. *N. Am. J. Fish. Manage.* **31**, 79-87.

Ewing, R.D., Ewing, G.S. (2002) Bimodal length distributions of cultured Chinook salmon and the relationship of length modes to adult survival. *Aquaculture* **209**, 139-155.

Farmer, G.J. (1994) Some factors which influence the survival of hatchery Atlantic salmon (*Salmo salar*) smolts utilized for enhancement purposes. *Aquaculture* **121**, 223-233.

Finstad, B., Iversen, M., Sandodden, R. (2003) Stress-reducing methods for releases of Atlantic salmon (*Salmo salar*) smolts in Norway. *Aquaculture* **222**, 203-214.

Finstad, B., Okland, F., Thorstad, E.B., Bjorn, P.A., McKinley, R.S. (2005) Migration of hatchery-reared Atlantic salmon and wild anadromous brown trout post-smolts in a Norwegian fjord system. *J. Fish Biol.* **66**, 86-96.

Fisher, J.P., Pearcy, W.C. (1987) Movements of coho, *Oncorhynchus kisutch* , and Chinook, *O. tshawytscha* , salmon tagged at sea off Oregon, Washington, and Vancouver Island during the summers 1982-85. *Fish. Bull.* **85**, 819-826.

Fisher, J.P., Pearcy, W.G. (1995) Distribution, migration, and growth of juvenile Chinook salmon, *Oncorhynchus tshawytscha*, off Oregon and Washington. *Fish. Bull.* **93**, 274-289.

Friedland, K.D., Hansen, L.P., Dunkley, D.A. (1998) Marine temperatures experienced by postsmolts and the survival of Atlantic salmon, *Salmo salar* L., in the North Sea area. *Fish. Oceanogr.* **7**, 22-34.

Friedland, K.D., Hansen, L.P., Dunkley, D.A., MacLean, J.C. (2000) Linkage between ocean climate, post-smolt growth, and survival of Atlantic salmon (*Salmo salar* L.) in the North Sea area. *ICES J. Mar. Sci.* **57**, 419-429.

Fukuwaka, M., Suzuki, T. (2002) Early sea mortality of mark-recaptured juvenile chum salmon in open coastal waters. *J. Fish Biol.* **60**, 3-12.

Fuss, H.J., Hopley, C.W. (2003) Gill Na+,K+-ATPase activity of hatchery chum salmon fry during freshwater rearing and acclimation to brackish water and its relationship to marine survival. *N. Am. J. Aquacult.* **65**, 134-140.

Gudjonsson, S., Jonsson, I.R., Antonsson, T. (2005) Migration of Atlantic salmon, *Salmo salar*, smolt through the estuary area of River Ellidaar in Iceland. *Environ. Biol. Fishes* **74**, 291-296.

Gunneroed, T.B., Hvidsten, N.A., Heggberget, T.G. (1988) Open sea releases of Atlantic salmon smolts, *Salmo salar* , in central Norway, 1973-83. *Can. J. Fish. Aquat. Sci.* **45**, 1340-1345.

Hall, J.E., Chamberlin, J., Kagley, A.N., Greene, C., Fresh, K.L. (2009) Effects of gastric and surgical insertions of dummy ultrasonic transmitters on juvenile Chinook salmon in seawater. *Trans. Am. Fish. Soc.* **138**, 52-57.

Halttunen, E., Rikardsen, A.H., Thorstad, E.B., Naesje, T.F., Jensen, J.L.A., Aas, O. (2010) Impact of catch-and-release practices on behavior and mortality of Atlantic salmon (*Salmo salar* L.) kelts. *Fish. Res.* **105**, 141-147.

Hamilton, K., Mysak, L.A. (1986) Possible effects of the Sitka eddy on sockeye (*Oncorhynchus nerka*) and pink salmon (*Oncorhynchus gorbuscha*) migration off southeast Alaska. *Can. J. Fish. Aquat. Sci.* **43**, 498-504.

Hansen, L.P. (2006) Migration and survival of farmed Atlantic salmon (*Salmo salar* L.) released from two Norwegian fish farms. *ICES J. Mar. Sci.* **63**, 1211-1217.

Hansen, L.P., Jacobsen, J.A. (2003) Origin and migration of wild and escaped farmed Atlantic salmon, *Salmo salar* L., in oceanic areas north of the Faroe Islands. *ICES J. Mar. Sci.* **60**, 110-119.

Hansen, L.P., Jonsson, B. (1991) The effect of timing of Atlantic salmon smolt and post-smolt release on the distribution of adult return. *Aquaculture* **98**, 61-67.

Hansen, L.P., Jonsson, N., Jonsson, B. (1993) Oceanic migration in homing Atlantic salmon. *Anim. Behav.* **45**, 927-941.

Hanson, K.C., Cooke, S.J., Hinch, S.G., Crossin, G.T., Patterson, D.A., English, K.K., Donaldson, M.R., Shrimpton, J.M., Van der Kraak, G., Farrell, A.P. (2008) Individual variation in migration speed of upriver-migrating sockeye salmon in the Fraser River in relation to their physiological and energetic status at marine approach. *Physiol. Biochem. Zool.* **81**, 255-268.

Hayes, S.A., Bond, M.H., Hanson, C.V., Freund, E.V., Smith, J.J., Anderson, E.C., Ammann, A.J., Macfarlane, R.B. (2008) Steelhead growth in a small central California watershed: Upstream and estuarine rearing patterns. *Trans. Am. Fish. Soc.* **137**, 114-128.

Hedger, R.D., Hatin, D., Dodson, J.J., Martin, F., Fournier, D., Caron, F., Whoriskey, F.G. (2009) Migration and swimming depth of Atlantic salmon kelts *Salmo salar* in coastal zone and marine habitats. *Mar. Ecol. Prog. Ser.* **392**, 179-192.

Hedger, R.D., Martin, F., Hatin, D., Caron, F., Whoriskey, F.G., Dodson, J.J. (2008) Active migration of wild Atlantic salmon *Salmo salar* smolt through a coastal embayment. *Mar. Ecol. Prog. Ser.* **355**, 235-246.

Heggberget, T.G., Oekland, F., Ugedal, O. (1993) Distribution and migratory behaviour of adult wild and farmed Atlantic salmon (*Salmo salar*) during return migration. *Aquaculture* **118**, 73-83.

Heintz, R.A., Rice, S.D., Wertheimer, A.C., Bradshaw, R.F., Thrower, F.P., Joyce, J.E., Short, J.W. (2000) Delayed effects on growth and marine survival of pink salmon *Oncorhynchus gorbuscha* after exposure to crude oil during embryonic development. *Mar. Ecol. Prog. Ser.* **208**, 205-216.

Hering, D.K., Bottom, D.L., Prentice, E.F., Jones, K.K., Fleming, I.A. (2010) Tidal movements and residency of subyearling Chinook salmon (*Oncorhynchus tshawytscha*) in an Oregon salt marsh channel. *Can. J. Fish. Aquat. Sci.* **67**, 524-533.

Hinke, J.T., Foley, D.G., Wilson, C., Watters, G.M. (2005a) Persistent habitat use by Chinook salmon *Oncorhynchus tshawytscha* in the coastal ocean. *Mar. Ecol. Prog. Ser.* **304**, 207-220.

Hinke, J.T., Watters, G.M., Boehlert, G.W., Zedonis, P. (2005b) Ocean habitat use in autumn by Chinook salmon in coastal waters of Oregon and California. *Mar. Ecol. Prog. Ser.* **285**, 181-192.

Hiramatsu, K., Ishida, Y. (1989) Random movement and orientation in pink salmon (*Oncorhynchus gorbuscha*) migrations. *Can. J. Fish. Aquat. Sci.* **46**, 1062-1066.

Holt, K.R., Cox, S.P., Sawada, J. (2009) In-season forecasting of coho salmon marine survival via coded wire tag recoveries. *N. Am. J. Fish. Manage.* **29**, 1165-1182.

Hubley, P.B., Amiro, P.G., Gibson, A.J.F., Lacroix, G.L., Redden, A.M. (2008) Survival and behaviour of migrating Atlantic salmon (*Salmo salar* L.) kelts in river, estuarine, and coastal habitat. *ICES J. Mar. Sci.* **65**, 1626-1634.

Hvidsten, N.A., Finstad, B., Kroglund, F., Johnsen, B.O., Strand, R., Arnekleiv, J.V., Bjorn, P.A. (2007) Does increased abundance of sea lice influence survival of wild Atlantic salmon post-smolt? *J. Fish Biol.* **71**, 1639-1648.

Hvidsten, N.A., Hansen, L.P. (1988) Increased recapture rate of adult Atlantic salmon, *Salmo salar* L., stocked as smolts at high water discharge. *J. Fish Biol.* **32**, 153-154.

Hvidsten, N.A., Johnsen, B.O.V.E. (1993) Increased recapture rate of adult Atlantic salmon released as smolts into large shoals of wild smolts in the River Orkla, Norway. *N. Am. J. Fish. Manage.* **13**, 272-276.

Hyun, S.Y., Myers, K.W., Talbot, A. (2007) Year-to-year variability in ocean recovery rate of Columbia River Upriver Bright fall Chinook salmon (*Oncorhynchus tshawytscha*). *Fish. Oceanogr.* **16**, 350-362.

Hyvarinen, P., Suuronen, P., Laaksonen, T. (2006) Short-term movements of wild and reared Atlantic salmon smolts in a brackish water estuary - preliminary study. *Fish. Manage. Ecol.* **13**, 399-401.

Ishida, Y., Yano, A., Ban, M., Ogura, M. (2001) Vertical movement of a chum salmon *Oncorhynchus keta* in the western North Pacific Ocean as determined by a depth-recording archival tag. *Fish. Sci.* **67**, 1030-1035.

Johnson, S.L., Power, J.H., Wilson, D.R., Ray, J. (2010) A comparison of the survival and migratory behavior of hatchery-reared and naturally reared steelhead smolts in the Alsea River and estuary, Oregon, using acoustic telemetry. *N. Am. J. Fish. Manage.* **30**, 55-71.

Jonsson, B., Jonsson, N., Hansen, L.P. (2007) Factors affecting river entry of adult Atlantic salmon in a small river. *J. Fish Biol.* **71**, 943-956.

Jonsson, N., Hansen, L.P., Jonsson, B. (1993) Migratory behaviour and growth of hatchery-reared post-smolt Atlantic salmon *Salmo salar*. *J. Fish Biol.* **42**, 435-443.

Jutila, E., Jokikokko, E., Ikonen, E. (2009) Post-smolt migration of Atlantic salmon, *Salmo salar* L., from the Simojoki river to the Baltic Sea. *J. Appl. Ichthyol.* **25**, 190-194.

Jutila, E., Jokikokko, E., Kallio-Nyberg, I., Saloniemi, I., Pasanen, P. (2003) Differences in sea migration between wild and reared Atlantic salmon (*Salmo salar* L.) in the Baltic Sea. *Fish. Res.* **60**, 333-343.

Kallio-Nyberg, I., Jutila, E., Jokikokko, E., Saloniemi, I. (2006) Survival of reared Atlantic salmon and sea trout in relation to marine conditions of smolt year in the Baltic Sea. *Fish. Res.* **80**, 295-304.

Kallio-Nyberg, I., Jutila, E., Saloniemi, I., Jokikokko, E. (2004) Association between environmental factors, smolt size and the survival of wild and reared Atlantic salmon from the Simojoki River in the Baltic Sea. *J. Fish Biol.* **65**, 122-134.

Kallio-Nyberg, I., Koljonen, M.L. (1997) The genetic consequence of hatchery-rearing on life-history traits of the Atlantic salmon (*Salmo salar* L): a comparative analysis of sea-ranched salmon with wild and reared parents. *Aquaculture* **153**, 207-224.

Kallio-Nyberg, I., Koljonen, M.L., Saloniemi, I. (2000) Effect of maternal and paternal line on spatial and temporal marine distribution in Atlantic salmon. *Anim. Behav.* **60**, 377-384.

Kallio-Nyberg, I., Peltonen, H., Rita, H. (1999) Effects of stock-specific and environmental factors on the feeding migration of Atlantic salmon (*Salmo salar*) in the Baltic Sea. *Can. J. Fish. Aquat. Sci.* **56**, 853-861.

Kallio-Nyberg, I., Salminen, M., Saloniemi, I., Kannala-Fisk, L. (2009) Marine Survival of reared Atlantic salmon in the Baltic Sea: The effect of smolt traits and annual factors. *Fish. Res.* **96**, 289-295.

Kallio-Nyberg, I., Salminen, M., Saloniemi, I., Lindroos, M. (2011a) Effects of marine survival, precocity and other life history traits on the cost-benefit of stocking salmon in the Baltic Sea. *Fish. Res.* **110**, 111-119.

Kallio-Nyberg, I., Saloniemi, I., Jutila, E., Jokikokko, E. (2011b) Effect of hatchery rearing and environmental factors on the survival, growth and migration of Atlantic salmon in the Baltic Sea. *Fish. Res.* **109**, 285-294.

Kallio-Nyberg, I., Saloniemi, I., Koljonen, M.L. (2007) Effects of parental and smolt traits on the marine survival of released Atlantic salmon (*Salmo salar*). *Aquaculture* **272**, 254-266.

Karppinen, P., Erkinaro, J., Niemela, E., Moen, K., Okland, F. (2004) Return migration of one-sea-winter Atlantic salmon in the River Tana. *J. Fish Biol.* **64**, 1179-1192.

Kennedy, B.M., Gale, W.L., Ostrand, K.G. (2007) Relationship between smolt gill Na+, K+ ATPase activity and migration timing to avian predation risk of steelhead trout (*Oncorhynchus mykiss*) in a large estuary. *Can. J. Fish. Aquat. Sci.* **64**, 1506-1516.

Kitahashi, T., Ando, H., Urano, A., Ban, M., Saito, S., Tanaka, H., Naito, Y., Ueda, H. (2000) Micro data logger analyses of homing behavior of chum salmon in Ishikari Bay. *Zool. Sci.* **17**, 1247-1253.

Kline, T.C., Willette, T.M. (2002) Pacific salmon (*Oncorhynchus* spp.) early marine feeding patterns based on N-15/N-14 and C-13/C-12 in Prince William Sound, Alaska. *Can. J. Fish. Aquat. Sci.* **59**, 1626-1638.

Labelle, M., Walters, C.J., Riddell, B. (1997) Ocean survival and exploitation of coho salmon (*Oncorhynchus kisutch*) stocks from the east coast of Vancouver Island, British Columbia. *Can. J. Fish. Aquat. Sci.* **54**, 1433-1449.

Lacroix, G.L., Knox, D., Stokesbury, M.J.W. (2005) Survival and behaviour of post-smolt Atlantic salmon in coastal habitat with extreme tides. *J. Fish Biol.* **66**, 485-498.

Lacroix, G.L., McCurdy, P. (1996) Migratory behaviour of post-smolt Atlantic salmon during initial stages of seaward migration. *J. Fish Biol.* **49**, 1086-1101.

Lacroix, G.L., McCurdy, P., Knox, D. (2004) Migration of Atlantic salmon postsmolts in relation to habitat use in a coastal system. *Trans. Am. Fish. Soc.* **133**, 1455-1471.

Linley, T.J. (2001) Influence of short-term estuarine rearing on the ocean survival and size at return of coho salmon in southeastern Alaska. *N. Am. J. Aquacult.* **63**, 306-311.

Lyse, A.A., Stefansson, S.O., Ferno, A. (1998) Behaviour and diet of sea trout post-smolts in a Norwegian fjord system. *J. Fish Biol.* **52**, 923-936.

Magnusson, A., Hilborn, R. (2003) Estuarine influence on survival rates of coho (*Oncorhynchus kisutch*) and Chinook salmon (*Oncorhynchus tshawytscha*) released from hatcheries on the US Pacific Coast. *Estuaries* **26**, 1094-1103.

Manel-la, N.P., Chittenden, C.M., Okland, F., Thorstad, E.B., Davidsen, J.G., Sivertsgard, R., McKinley, R.S., Finstad, B. (2011) Does river of origin influence the early marine migratory performance of *Salmo salar*? *J. Fish Biol.* **78**, 624-634.

Manel-La, N.P., Thorstad, E.B., Davidsen, J.G., Okland, F., Sivertsgard, R., McKinley, R.S., Finstad, B. (2009) Vertical movements of Atlantic salmon post-smolts relative to measures of salinity and water temperature during the first phase of the marine migration. *Fish. Manage. Ecol.* **16**, 147-154.

Martin, F., Hedger, R.D., Dodson, J.J., Fernandes, L., Hatin, D., Caron, F., Whoriskey, F.G. (2009) Behavioural transition during the estuarine migration of wild Atlantic salmon (*Salmo salar* L.) smolt. *Ecol. Freshwat. Fish* **18**, 406-417.

McMichael, G.A., Eppard, M.B., Carlson, T.J., Carter, J.A., Ebberts, B.D., Brown, R.S., Weiland, M., Ploskey, G.R., Harnish, R.A., Deng, Z.D. (2010) The juvenile salmon acoustic telemetry system: a new tool. *Fisheries* **35**, 9-22.

Melnychuk, M.C. (2009) Estimation of survival and detection probabilities for multiple tagged salmon stocks with nested migration routes, using a large-scale telemetry array. *Marine and Freshwater Research* **60**, 1231-1243.

Melnychuk, M.C., Welch, D.W., Walters, C.J. (2010) Spatio-temporal migration patterns of Pacific salmon smolts in rivers and coastal marine waters. *Plos One* **5**, e12916. doi:12910.11371/journal.pone.0012916.

Melnychuk, M.C., Welch, D.W., Walters, C.J., Christensen, V. (2007) Riverine and early ocean migration and mortality patterns of juvenile steelhead trout (*Oncorhynchus mykiss*) from the Cheakamus River, British Columbia. *Hydrobiologia* **582**, 55-65.

Miller, K.M., Li, S., Kaukinen, K.H., Ginther, N., Hammill, E., Curtis, J.M.R., Patterson, D.A., Sierociniski, T., Donnison, L., Pavlidis, P., Hinch, S.G., Hruska, K.A., Cooke, S.J., English, K.K., Farrell, A.P. (2011) Genomic signatures predict migration and spawning failure in wild Canadian salmon. *Science* **331**, 214-217.

Moffett, I.J.J., Crozier, W.W., Kennedy, G.J.A. (1997) A comparison of five external marks for Atlantic salmon, *Salmo salar* L. *Fish. Manage. Ecol.* **4**, 49-53.

Montevecchi, W.A., Cairns, D.K., Birt, V.L. (1988) Migration of postsmolt Atlantic salmon, *Salmo salar*, off northeastern Newfoundland, as inferred by tag recoveries in a seabird colony. *Can. J. Fish. Aquat. Sci.* **45**, 568-571.

Moore, A., Cotter, D., Quayle, V., Rogan, G., Poole, R., Lower, N., Privitera, L. (2008) The impact of a pesticide on the physiology and behaviour of hatchery-reared Atlantic salmon, *Salmo salar*, smolts during the transition from fresh water to the marine environment. *Fish. Manage. Ecol.* **15**, 385-392.

Moore, A., Ives, S., Mead, T.A., Talks, L. (1998) The migratory behaviour of wild Atlantic salmon (*Salmo salar* L.) smelts in the River Test and Southampton Water, southern England. *Hydrobiologia* **372**, 295-304.

Moore, A., Potter, E.C.E., Milner, N.J., Bamber, S. (1995) The migratory behavior of wild Atlantic salmon (*Salmo salar*) smolts in the estuary of the River Conwy, North Wales. *Can. J. Fish. Aquat. Sci.* **52**, 1923-1935.

Moore, M.E., Berejikian, B.A., Tezak, E.P. (2010a) Early Marine survival and behavior of steelhead trout (*Oncorhynchus mykiss*) smolts through Hood Canal and the Strait of Juan de Fuca. *Trans. Am. Fish. Soc* **139**.

Moore, M.E., Goetz, F.A., Van Doornik, D.M., Tezak, E.P., Quinn, T.P., Reyes-Tomassini, J.J., Berejikian, B.A. (2010b) Early marine migration patterns of wild coastal cutthroat trout (*Oncorhynchus clarki clarki*), steelhead trout (*Oncorhynchus mykiss*), and their hybrids. *Plos One* **5**, 1-10.

Mortensen, D., Wertheimer, A., Taylor, S., Landingham, J. (2000) The relation between early marine growth of pink salmon, *Oncorhynchus gorbuscha*, and marine water temperature, secondary production, and survival to adulthood. *Fish. Bull.* **98**, 319-335.

Moser, M.L., Olson, A.F., Quinn, T.P. (1991) Riverine and estuarine migratory behavior of coho salmon (*Oncorhynchus kisutch*) smolts. *Can. J. Fish. Aquat. Sci.* **48**, 1670-1678.

Newman, K. (1997) Bayesian averaging of generalized linear models for passive integrated transponder tag recoveries from salmonids in the Snake River. *N. Am. J. Fish. Manage.* **17**, 362-377.

Newman, K.B. (1998) State-space modeling of animal movement and mortality with application to salmon. *Biometrics* **54**, 1290-1314.

Newman, K.B. (2000) Hierarchic modeling of salmon harvest and migration. *Journal of Agricultural Biological and Environmental Statistics* **5**, 430-455.

Newman, K.B., Rice, J. (2002) Modeling the survival of Chinook salmon smolts outmigrating through the lower Sacramento River system. *Journal of the American Statistical Association* **97**, 983-993.

Nielsen, J.L., Turner, S.M., Zimmerman, C.E. (2011) Electronic tags and genetics explore variation in migrating steelhead kelts (*Oncorhynchus mykiss*), Ninilchik River, Alaska. *Can. J. Fish. Aquat. Sci.* **68**, 1-16.

Ogura, M. (1999) Rapid dives and ascents of sockeye salmon *Oncorhynchus nerka* observed by ultrasonic telemetry in the open sea. *Fish. Sci.* **65**, 659-660.

Ogura, M., Ishida, Y. (1992) Swimming behavior of coho salmon, *Oncorhynchus kisutch*, in the open sea as determined by ultrasonic telemetry. *Can. J. Fish. Aquat. Sci.* **49**, 453-457.

Ogura, M., Ishida, Y. (1995) Homing behavior and vertical movements of 4 species of Pacific salmon (*Oncorhynchus* spp) in the Central Bering Sea. *Can. J. Fish. Aquat. Sci.* **52**, 532-540.

Olson, A.F., Quinn, T.P. (1993) Vertical and horizontal movements of adult Chinook salmon *Oncorhynchus tshawytscha* in the Columbia River Estuary. *Fish. Bull.* **91**, 171-178.

Orsi, J.A., Jaenicke, H.W. (1996) Marine distribution and origin of prerecruit Chinook salmon, *Oncorhynchus tshawytscha*, in southeastern Alaska. *Fish. Bull.* **94**, 482-497.

Pascual, M.A., Quinn, T.P. (1991) Evaluation of alternative models of the coastal migration of adult Fraser River sockeye salmon (*Oncorhynchus nerka*). *Can. J. Fish. Aquat. Sci.* **48**, 799-810.

Pedersen, S., Rasmussen, G., Nielsen, E.E., Karlsson, L., Nyberg, P. (2007) Straying of Atlantic salmon, *Salmo salar*, from delayed and coastal releases in the Baltic Sea, with special focus on the Swedish west coast. *Fish. Manage. Ecol.* **14**, 21-32.

Pinder, A.C., Riley, W.D., Ibbotson, A.T., Beaumont, W.R.C. (2007) Evidence for an autumn downstream migration and the subsequent estuarine residence of 0+year juvenile Atlantic salmon *Salmo salar* L., in England. *J. Fish Biol.* **71**, 260-264.

Quinn, T.P. (1988) Estimated swimming speeds of migrating adult sockeye salmon. *Canadian Journal of Zoology* **66**, 2160-2163.

Quinn, T.P., Dickerson, B.R., Vollestad, L.A. (2005) Marine survival and distribution patterns of two Puget Sound hatchery populations of coho (*Oncorhynchus kisutch*) and Chinook (*Oncorhynchus tshawytscha*) salmon. *Fish. Res.* **76**, 209-220.

Quinn, T.P., Peterson, N.P. (1996) The influence of habitat complexity and fish sire on over-winter survival and growth of individually marked juvenile coho salmon (*Oncorhynchus kisutch*) in Big Beef creek, Washington. *Can. J. Fish. Aquat. Sci.* **53**, 1555-1564.

Quinn, T.P., Terhart, B.A., Groot, C. (1989) Migratory orientation and vertical movements of homing adult sockeye salmon, *Oncorhynchus nerka*, in coastal waters. *Anim Behav* **37**, 587-599.

Quinn, T.P., Unwin, M.J., Kinnison, M.T. (2011) Contemporary divergence in migratory timing of naturalized populations of Chinook salmon, *Oncorhynchus tshawytscha*, in New Zealand. *Evol. Ecol. Res.* **13**, 45-54.

Rechisky, E.L., Welch, D.W., Porter, A.D., Jacobs, M.C., Ladouceur, A. (2009) Experimental measurement of hydrosystem-induced delayed mortality in juvenile Snake River spring Chinook salmon (*Oncorhynchus tshawytscha*) using a large-scale acoustic array. *Can. J. Fish. Aquat. Sci.* **66**, 1019-1024.

Reddin, D.G., Downton, P., Fleming, I.A., Hansen, L.P., Mahon, A. (2011) Behavioural ecology at sea of Atlantic salmon (*Salmo salar* L.) kelts from a Newfoundland (Canada) river. *Fish. Oceanogr.* **20**, 174-191.

Reddin, D.G., Downton, P., Friedland, K.D. (2006) Diurnal and nocturnal temperatures for Atlantic salmon postsmolts (*Salmo salar* L.) during their early marine life. *Fish. Bull.* **104**, 415-427.

Reddin, D.G., Friedland, K.D., Downton, P., Dempson, J.B., Mullins, C.C. (2004) Thermal habitat experienced by Atlantic salmon (*Salmo salar* L.) kelts in coastal Newfoundland waters. *Fish. Oceanogr.* **13**, 24-35.

Rice, C.A., Greene, C.M., Moran, P., Teel, D.J., Kuligowski, D.R., Reisenbichler, R.R., Beamer, E.M., Karr, J.R., Fresh, K.L. (2011) Abundance, stock origin, and length of marked and unmarked juvenile Chinook salmon in the surface waters of greater Puget Sound. *Trans. Am. Fish. Soc.* **140**, 170-189.

Ruggerone, G.T., Goetz, F.A. (2004) Survival of Puget Sound Chinook salmon (*Oncorhynchus tshawytscha*) in response to climate-induced competition with pink salmon (*Oncorhynchus gorbuscha*). *Can. J. Fish. Aquat. Sci.* **61**, 1756-1770.

Russell, I.C., Moore, A., Ives, S., Kell, L.T., Ives, M.J., Stonehewer, R.O. (1998) The migratory behaviour of juvenile and adult salmonids in relation to an estuarine barrage. *Hydrobiologia* **372**, 321-333.

Ryding, K.E., Skalski, J.R. (1999) Multivariate regression relationships between ocean conditions and early marine survival of coho salmon (*Oncorhynchus kisutch*). *Can. J. Fish. Aquat. Sci.* **56**, 2374-2384.

Salminen, M. (1997) Relationships between smolt size, postsmolt growth and sea age at maturity in Atlantic salmon ranched in the Baltic Sea. *J. Appl. Ichthyol.* **13**, 121-130.

Salminen, M., Kuikka, S. (1995) Annual variability in survival of sea-ranched Baltic salmon, *Salmo salar* L.: significance of smolt size and marine conditions. *Fish. Manage. Ecol.* **2**, 171-184.

Saloniemi, I., Jokikokko, E., Kallio-Nyberg, I., Jutila, E., Pasanen, P. (2004) Survival of reared and wild Atlantic salmon smolts: size matters moire in bad years. *ICES J. Mar. Sci.* **61**, 782-787.

Seeb, L.W., Crane, P.A. (1999) Allozymes and mitochondrial DNA discriminate Asian and North American populations of chum salmon in mixed-stock fisheries along the south coast of the Alaska Peninsula. *Trans. Am. Fish. Soc.* **128**, 88-103.

Seeb, L.W., Crane, P.A., Kondzela, C.M., Wilmot, R.L., Urawa, S., Varnavskaya, N.V., Seeb, J.E. (2004) Migration of Pacific Rim chum salmon on the high seas: insights from genetic data. *Environ. Biol. Fishes* **69**, 21-36.

Seeb, L.W., Seeb, J.E., Habicht, C., Farley, E.V., Jr., Utter, F.M. (2011) Single-nucleotide polymorphic genotypes reveal patterns of early juvenile migration of sockeye salmon in the eastern Bering Sea. *Trans. Am. Fish. Soc.* **140**, 734-748.

Semmens, B.X. (2008) Acoustically derived fine-scale behaviors of juvenile Chinook salmon (*Oncorhynchus tshawytscha*) associated with intertidal benthic habitats in an estuary. *Can. J. Fish. Aquat. Sci.* **65**, 2053-2062.

Serrano, I., Rivinoja, P., Karlsson, L., Larsson, S. (2009) Riverine and early marine survival of stocked salmon smolts, *Salmo salar* L., descending the Testebo River, Sweden. *Fish. Manage. Ecol.* **16**, 386-394.

Shelton, R.G.J., Turrell, W.R., Macdonald, A., McLaren, I.S., Nicoll, N.T. (1997) Records of post-smolt Atlantic salmon, *Salmo salar* L, in the Faroe-Shetland Channel in June 1996. *Fish. Res.* **31**, 159-162.

Siira, A., Suuronen, P., Kreivi, P., Erkinaro, J. (2006) Size of wild and hatchery-reared Atlantic salmon populations in the northern Baltic Sea estimated by a stratified mark-recapture method. *ICES J. Mar. Sci.* **63**, 1477-1487.

Sivertsgard, R., Thorstad, E.B., Okland, F., Finstad, B., Bjorn, P.A., Jepsen, N., Nordal, T., McKinley, R.S. (2007) Effects of salmon lice infection and salmon lice protection on fjord migrating Atlantic salmon and brown trout post-smolts. *Hydrobiologia* **582**, 35-42.

Skilbrei, O.T. (2010a) Adult recaptures of farmed Atlantic salmon post-smolts allowed to escape during summer. *Aquaculture Environment Interactions* **1**, 147-153.

Skilbrei, O.T. (2010b) Reduced migratory performance of farmed Atlantic salmon post-smolts from a simulated escape during autumn. *Aquaculture Environment Interactions* **1**, 117-125.

Skilbrei, O.T., Holst, J.C., Asplin, L., Mortensen, S. (2010) Horizontal movements of simulated escaped farmed Atlantic salmon (*Salmo salar*) in a western Norwegian fjord. *ICES J. Mar. Sci.* **67**, 1206-1215.

Skilbrei, O.T., Wennevik, V. (2006) Survival and growth of sea-ranched Atlantic salmon, *Salmo salar* L., treated against sea lice before release. *ICES J. Mar. Sci.* **63**, 1317-1325.

Smith, G.W., Smith, I.P., Armstrong, S.M. (1994) The relationship between river flow and entry to the Aberdeenshire-Dee by returning adult Atlantic salmon. *J. Fish Biol.* **45**, 953-960.

Smith, I.P., Smith, G.W. (1997) Tidal and diel timing of river entry by adult Atlantic salmon returning to the Aberdeenshire Dee, Scotland. *J. Fish Biol.* **50**, 463-474.

Solazzi, M.F., Nickelson, T.E., Johnson, S.L. (1991) Survival, contribution, and return of hatchery coho salmon (*Oncorhynchus kisutch*) released into fresh-water, estuarine, and marine environments. *Can. J. Fish. Aquat. Sci.* **48**, 248-253.

Solomon, D.J., Sambrook, H.T. (2004) Effects of hot dry summers on the loss of Atlantic salmon, *Salmo salar*, from estuaries in South West England. *Fish. Manage. Ecol.* **11**, 353-363.

Stasko, A.B. (1975) Progress of migrating Atlantic salmon (Salmo salar) along an estuary, observed by ultrasonic tracking. *J. Fish Biol.* **7**, 329-338.

Stasko, A.B., Horrall, R.M., Hasler, A.D. (1976) Coastal movements of adult Fraser River sockeye salmon (*Oncorhynchus nerka*) observed by ultrasonic tracking. *Trans. Am. Fish. Soc.* **105**, 64-71.

Staurnes, M., Lysfjord, G., Hansen, L.P., Heggberget, T.G. (1993) Recapture rates of hatchery-reared Atlantic salmon (*Salmo salar*) related to smolt development and time of release. *Aquaculture* **118**, 327-337.

Sweeting, R.M., Beamish, R.J., Noakes, D.J., Neville, C.M. (2003) Replacement of wild coho salmon by hatchery-reared coho salmon in the Strait of Georgia over the past three decades. *N. Am. J. Fish. Manage.* **23**, 492-502.

Tanaka, H., Takagi, Y., Naito, Y. (2000) Behavioural thermoregulation of chum salmon during homing migration in coastal waters. *J. Exp. Biol.* **203**, 1825-1833.

Tanaka, H., Takagi, Y., Naito, Y. (2001) Swimming speeds and buoyancy compensation of migrating adult chum salmon *Oncorhynchus keta* revealed by speed/depth/acceleration data logger. *J. Exp. Biol.* **204**, 3895-3904.

Teo, S.L.H., Botsford, L.W., Hastings, A. (2009) Spatio-temporal covariability in coho salmon (*Oncorhynchus kisutch*) survival, from California to southeast Alaska. *Deep Sea Res. Part II* **56**, 2570-2578.

Thorstad, E.B., Okland, F., Finstad, B., Sivertsgard, R., Bjorn, P.A., McKinley, R.S. (2004) Migration speeds and orientation of Atlantic salmon and sea trout post-smolts in a Norwegian fjord system. *Environ. Biol. Fishes* **71**, 305-311.

Thorstad, E.B., Okland, F., Finstad, B., Sivertsgard, R., Plantalech, N., Bjorn, P.A., McKinley, R.S. (2007) Fjord migration and survival of wild and hatchery-reared Atlantic salmon and wild brown trout post-smolts. *Hydrobiologia* **582**, 99-107.

Thorstad, E.B., Uglem, I., Arechavala-Lopez, P., Okland, F., Finstad, B. (2011) Low survival of hatchery-released Atlantic salmon smolts during initial river and fjord migration. *Boreal Environ. Res.* **16**, 115-120.

Tipping, J.M. (2008) The effect of juvenile length in autumn on 2-year-old male and adult coho salmon survival rates at three Washington hatcheries. *N. Am. J. Aquacult.* **70**, 111-114.

Ueda, H. (2011) Physiological mechanism of homing migration in Pacific salmon from behavioural to molecular biological approaches. *Gen. Comp. Endocrinol.* **170**, 222-232.

Ueno, Y. (1991) Migration of mature chum salmon along Iwate-Prefecture Coast estimated by tagging experiments. *Nippon Suisan Gakkaishi* **57**, 1445-1452.

Ueno, Y., Hiramatsu, K. (1990) A method for estimating mortality coefficents of chum salmon *Oncorhynchus keta* from tagging experiments. *Nippon Suisan Gakkaishi* **56**, 1079-1082.

Unwin, M.J. (1997) Fry-to-adult survival of natural and hatchery-produced Chinook salmon (*Oncorhynchus tshawytscha*) from a common origin. *Can. J. Fish. Aquat. Sci.* **54**, 1246-1254.

Unwin, M.J., Glova, G.J. (1997) Changes in life history parameters in a naturally spawning population of Chinook salmon (*Oncorhynchus tshawytscha*) associated with releases of hatchery-reared fish. *Can. J. Fish. Aquat. Sci.* **54**, 1235-1245.

Unwin, M.J., Kinnison, M.T., Boustead, N.C., Quinn, T.P. (2003) Genetic control over survival in Pacific salmon (*Oncorhynchus* spp.): experimental evidence between and within populations of New Zealand Chinook salmon (*O. tshawytscha*). *Can. J. Fish. Aquat. Sci.* **60**, 1-11.

Virtanen, E., Soderholmtana, L., Soivio, A., Forsman, L., Muona, M. (1991) Effect of physiological condition and smoltification status at smolt release on subsequent catches of adult salmon. *Aquaculture* **97**, 231-257.

Voegeli, F.A., Lacroix, G.L., Anderson, J.M. (1998) Development of miniature pingers for tracking Atlantic salmon smolts at sea. *Hydrobiologia* **372**, 35-46.

Walker, R.V., Myers, K.W., Davis, N.D., Aydin, K.Y., Friedland, K.D., Carlson, H.R., Boehlert, G.W., Urawa, S., Ueno, Y., Anma, G. (2000) Diurnal variation in thermal environment experienced by salmonids in the North Pacific as indicated by data storage tags. *Fish. Oceanogr.* **9**, 171-186.

Walter, E.E., Scandol, J.P., Healey, M.C. (1997) A reappraisal of the ocean migration patterns of Fraser River sockeye salmon (*Oncorhynchus nerka*) by individual-based modelling. *Can. J. Fish. Aquat. Sci.* **54**, 847-858.

Wattersi, G.A., Bessey, C. (2008) Variation in the probability that male coho salmon will mature early: Inferences from hierarchical models. *Trans. Am. Fish. Soc.* **137**, 70-95.

Weitkamp, L., Neely, K. (2002) Coho salmon (*Oncorhynchus kisutch*) ocean migration patterns: insight from marine coded-wire tag recoveries. *Can. J. Fish. Aquat. Sci.* **59**, 1100-1115.

Weitkamp, L.A. (2010) Marine distributions of Chinook salmon from the west coast of North America determined by coded wire tag recoveries. *Trans. Am. Fish. Soc.* **139**, 147-170.

Welch, D.W., Melnychuk, M.C., Payne, J.C., Rechisky, E.L., Porter, A.D., Jackson, G.D., Ward, B.R., Vincent, S.P., Wood, C.C., Semmens, J. (2011) In situ measurement of coastal ocean movements and survival of juvenile Pacific salmon. *Proceedings of the Academy of Natural Sciences USA* **108**, 8708-8713.

Welch, D.W., Melnychuk, M.C., Rechisky, E.R., Porter, A.D., Jacobs, M.C., Ladouceur, A., McKinley, R.S., Jackson, G.D. (2009) Freshwater and marine migration and survival of endangered Cultus Lake sockeye salmon (*Oncorhynchus nerka*) smolts using POST, a large-scale acoustic telemetry array. *Can. J. Fish. Aquat. Sci.* **66**, 736-750.

Whoriskey, F.G., Brooking, P., Doucette, G., Tinker, S., Carr, J.W. (2006) Movements and survival of sonically tagged farmed Atlantic salmon released in Cobscook Bay, Maine, USA. *ICES J. Mar. Sci.* **63**, 1218-1223.

Wilkins, N.P., Cotter, D., O'Maoileidigh, N. (2001) Ocean migration and recaptures of tagged, triploid, mixed-sex and all-female Atlantic salmon (*Salmo salar* L.) released from rivers in Ireland. *Genetica* **111**, 197-212.

Withler, R.E., Beacham, T.D., Solar, II, Donaldson, E.M. (1995) Freshwater growth, smelting, and marine survival and growth of diploid and triploid coho salmon (*Oncorhynchus kisutch*). *Aquaculture* **136**, 91-107.

Yano, A., Ogura, M., Sato, A., Sakaki, Y., Ban, M., Nagasawa, K. (1996) Development of ultrasonic telemetry technique for investigating the magnetic sense of salmonids. *Fish. Sci.* **62**, 698-704.

Yano, K., Nakamura, A. (1992) Observations on the effect of visual and olfactory ablation on the swimming behavior of migrating adult chum salmon, *Oncorhynchus keta*. *Jap. J. Ichthyol.* **39**, 67-83.
